# Physiologically Achievable Concentration of 2-Deoxy-D-Glucose Stimulates IFN-γ Secretion in Activated T Cells In Vitro

**DOI:** 10.3390/ijms251910384

**Published:** 2024-09-26

**Authors:** Jernej Repas, Tjaša Frlic, Tadeja Snedec, Andreja Nataša Kopitar, Harald Sourij, Andrej Janež, Mojca Pavlin

**Affiliations:** 1 Institute of Biophysics, Faculty of Medicine, University of Ljubljana, 1000 Ljubljana, Slovenia; jernej.repas@mf.uni-lj.si (J.R.); tjasa.frlic@mf.uni-lj.si (T.F.); tadeja.snedec@mf.uni-lj.si (T.S.); 2 Institute of Microbiology and Immunology, Faculty of Medicine, University of Ljubljana, 1000 Ljubljana, Slovenia; andreja-natasa.kopitar@mf.uni-lj.si; 3 Trials Unit for Interdisciplinary Metabolic Medicine, Division of Endocrinology and Diabetology, Medical University Graz, 8010 Graz, Austria; ha.sourij@medunigraz.at; 4 Clinical Department of Endocrinology, Diabetes and Metabolic Diseases, University Medical Centre Ljubljana, 1000 Ljubljana, Slovenia; andrej.janez@kclj.si; 5 Group for Nano- and Biotechnological Applications, Faculty of Electrical Engineering, University of Ljubljana, 1000 Ljubljana, Slovenia

**Keywords:** T cells, interferon gamma, 2-deoxy-D-glucose, mitochondria, ER stress, protein N-glycosylation, CAR T

## Abstract

2-deoxy-D-glucose (2DG) is a glycolysis and protein N-glycosylation inhibitor with promising anti-tumor and immunomodulatory effects. However, 2DG can also suppress T cell function, including IFN-γ secretion. Few human T cell studies have studied low-dose 2DG, which can increase IFN-γ in a Jurkat clone. We therefore investigated 2DG’s effect on IFN-γ in activated human T cells from PBMCs, with 2DG treatment commenced either concurrently with activation or 48 h after activation. Concurrent 2DG treatment decreased IFN-γ secretion in a dose-dependent manner. However, 2DG treatment of pre-activated T cells had a hormetic effect on IFN-γ, with 0.15–0.6 mM 2DG (achievable in vivo) increasing and >2.4 mM 2DG reducing its secretion. In contrast, IL-2 levels declined monotonously with increasing 2DG concentration. Lower 2DG concentrations reduced PD-1 and increased CD69 expression regardless of treatment timing. The absence of increased T-bet or Eomes expression or IFNG transcription suggests another downstream mechanism. 2DG dose-dependently induced the unfolded protein response, suggesting a possible role in increased IFN-γ secretion, possibly by increasing the ER folding capacity for IFN-γ via increased chaperone expression. Overall, low-dose, short-term 2DG exposure could potentially improve the T cell anti-tumor response.

## 1. Introduction

T cell-focused immunotherapy has emerged as the most prominent breakthrough in cancer treatment in recent years. The use of genetically engineered chimeric antigen receptor (CAR) T cells and immune checkpoint inhibitors (ICI) targeting cytotoxic T-lymphocyte associated protein 4 (CTLA4) or programmed death 1 (PD1)/programmed death-ligand 1 (PD-L1) axis has drastically improved survival in several cancer types. Additionally, the anti-tumor immune response has been recognized as a key factor in successful chemotherapy or radiotherapy [1]. Nevertheless, a significant part of cancer patients do not respond to ICI therapy, leading to a continuing search for ways to improve anti-tumor T cell responses [2]. One promising approach has focused on the metabolism of tumor cells, T cells and their interplay in the tumor microenvironment [3,4,5]. Increased aerobic glycolysis is a cardinal feature of most cancer cell types [6,7]. In addition to supporting cancer cell proliferation [7], it can also lead to glucose depletion and the suppression of T cell function in the hypoxic tumor microenvironment especially in conjunction with PD-1/PD-L1 signaling [3]. Targeting the metabolism of tumor cells and T cells has therefore been suggested as a possible approach to improve T cell anti-tumor immunity [8,9,10].

One promising metabolic inhibitor under investigation for its anti-tumor and immunomodulatory effects is 2-deoxy-D-glucose (2DG) [11,12]. As a structural analog of glucose, it is a competitive glycolysis inhibitor. Its phosphorylation by hexokinase yields 2DG-6-phosphate, which inhibits glucose-6-phosphate isomerase [13]. Hexokinase inhibition requires mM 2DG concentrations, so lower 2DG concentrations can increase the pentose phosphate pathway (PPP) flux [13] and thus decrease reactive oxygen species (ROS) levels. 2DG, as a structural analog of mannose, also competitively inhibits its incorporation into dolichol-linked oligosaccharides, thereby disrupting protein N-glycosylation in the endoplasmic reticulum (ER) [14,15,16,17]. The resulting ER stress and unfolded protein response (UPR) can lead to the activation of adenosine monophosphate-activated protein kinase (AMPK) and autophagy [18,19], resulting in cancer cell apoptosis when unresolved [16,20,21,22].

Due to these effects, 2DG has been investigated as a promising anti-cancer drug [11], especially in combination with other metabolic inhibitors such as metformin [23,24,25,26,27,28], or as an adjuvant to chemotherapy [29,30,31,32,33,34] or radiotherapy [35,36]. Several clinical trials have demonstrated the safety and tolerability of 2DG in patients [32,37]. Moreover, recent studies also indicate that 2DG may indirectly improve anti-tumor immunity by increasing immunogenic cell death in combination with chemotherapy [34] and reducing the glycosylation and surface expression of inhibitory immune checkpoints PD-L1 [38,39,40] and PD-1 [40,41,42] on cancer cells and T cells, respectively. The altered glycosylation of cancer cell surface antigens by 2DG on can also improve the effectiveness of CAR T therapy [43].

However, the direct effects of 2DG on T cell function are still not entirely clear. 2DG as a glycolysis inhibitor could also suppress anti-tumor T cell responses as glycolysis supports the fast proliferation of activated T cells [44,45] and is crucial for several effector functions, particularly the secretion of interferon gamma (IFN-γ) [46,47,48,49]. Glycolysis enables IFN-γ translation by preventing glyceraldehyde-3-phosphate dehydrogenase (GAPDH) binding to its mRNA [48] and increasing its transcription [49]. It was shown that 2DG treatment of T cells during activation can reduce IFN-γ secretion in vitro [46,50,51,52] and in rodent models in vivo [53,54,55], suggesting that 2DG could adversely affect the IFN-γ production in the context of the anti-tumor immune response. On the other hand, 2DG treatment of T cells ex vivo prior to adoptive transfer can improve the outcomes of adoptive T cell [56,57] or, more recently, CAR T cell therapy [42]. For example, a recent report found that a two-week 2DG treatment of activated human T cells in the presence of exogenous IL-2 increased their natural killer-like properties, including increased cytotoxicity and IFN-γ secretion, by interfering with protein N-glycosylation [57].

This raises the question whether 2DG could also have a direct stimulatory effect on T cell function, particularly IFN-γ secretion, when used as an adjuvant cancer treatment. Previous in vitro studies with human T cells often used relatively high (≥2 mM) 2DG concentrations, whereas only 0.6 mM is achievable in the blood of patients [32,58]. While this is not an issue for ex vivo treatment during adoptive (e.g., CAR T) therapy, it is essential to investigate the effects of low, physiologically achievable concentrations of 2DG for any potential use in vivo. In addition to the concentration, both 2DG treatment duration and its timing in relation to T cell activation (before, after or concurrent with activation) could play a role in the effect of 2DG and help explain the somewhat contradictory results of 2DG on T cells in the published literature. Intriguingly, we have previously found that even a short (24 h) 0.6 mM 2DG treatment can increase IFN-γ secretion in one clone of activated Jurkat cells used as a T cell model [40].

In the present paper, we investigate the effect of a low, in vivo achievable concentration of 2DG on IFN-γ secretion in primary T cells from peripheral blood mononuclear cells (PBMCs). We investigated the effect of 2DG as both a function of 2DG concentration and treatment timing relative to T cell activation. Additionally, we study several mechanisms that are potentially responsible for increased IFN-γ secretion by a low concentration of 2DG.

## 2. Results

### 2.1. 2-Deoxy-D-Glucose Treatment of Pre-Activated T Cells from PBMC Displays a Hormetic Effect on IFN-γ Secretion

**Figure 1:**
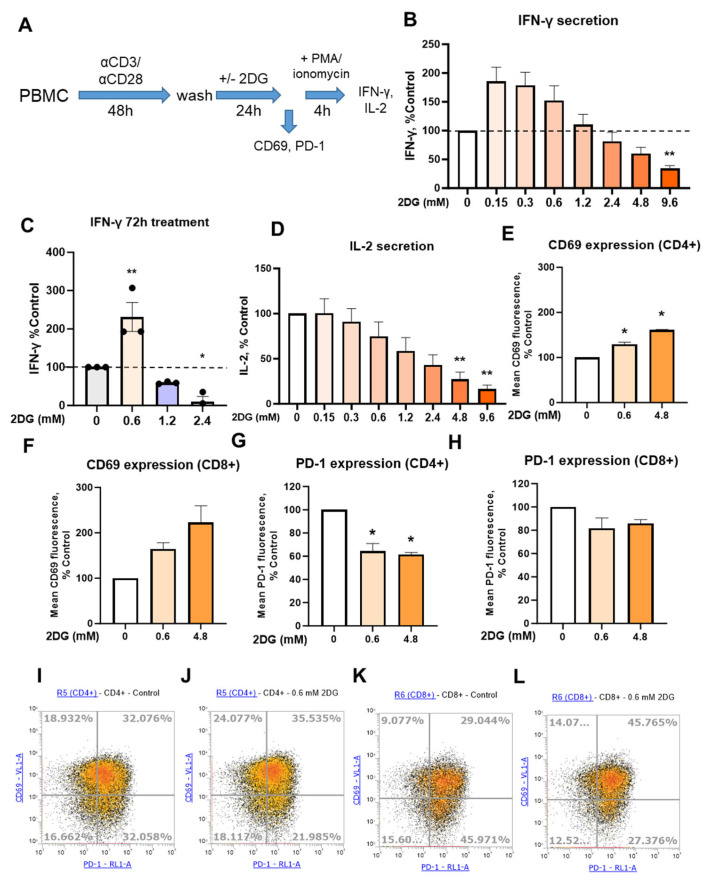
The effect of 2DG treatment on cytokine secretion, T cell activation and PD-1 expression in pre-activated T cells from PBMC. PBMC were activated with anti-CD3 and anti-CD28 antibodies for 48 h (72 h in (**C**)), harvested and washed, then treated for 24 h (72 h in (**C**)) with 0.6 mM or 4.8 mM 2DG (denoted by shades of orange), stained with antibodies and analyzed with flow cytometry. For cytokine secretion, the cells were restimulated after the treatment with PMA and ionomycin for 4 h, after which media supernatants were collected (**A**). The concentration of IFN-γ (**B**,**C**) and IL-2 (**D**) in supernatants were determined with ELISA. The expression levels of activation marker CD69 (**E**,**F**) and exhaustion marker/immune checkpoint PD-1 (**G**,**H**) were determined in CD4+ (**E**,**G**) and CD8+ (**F**,**H**) T cells using flow cytometry. The gating strategy and raw data are shown in Supplementary Figure S1. The data represent the mean ± SEM of three (**C**,**E**–**G**) to four (**B**,**D**) independent experiments with different healthy donors. * *p* < 0.05, ** *p* < 0.01 as determined by repeated measures one-way ANOVA with Dunnett’s post-hoc test. (**I**–**L**) Representative density plots for PD-1+/- CD69+/- populations are shown for control (**I**,**L**) or 0.6 mM 2DG treated (**J**,**L**) CD4+ (**I**,**J**) and CD8+ (**K**,**L**) T cells.

2DG has been shown to affect the expression of both PD-1 and PD-L1 which could be beneficial in the context of anti-tumor T cell immunity [38,39,40,41]. At the same time, 2DG was shown to have immunosuppressive effects on IFN-γ secretion in rodents [53,54,55] and in vitro T cell studies [50,51,52]. Since in vitro studies in particular often use high (>2 mM) 2DG concentrations, we first investigated how 2DG affects IFN-γ secretion in primary T cells at low (≤0.6 mM) concentration achievable in the plasma of patients versus a higher (≥2.4 mM) 2DG concentration where a more pronounced block of glycolysis is observed. In order to exclude the potential effect of 2DG treatment on initial T cell activation [50], T cells from PBMC of healthy donors were first activated for 48 h without the presence of 2DG, followed by 24 h 2DG treatment and subsequent 4 h restimulation with phorbol 12-myristate 13-acetate (PMA) and ionomycin (Figure 1A).

We found that 2DG exhibited a dose-dependent increase in IFN-γ secretion (ns) with a maximal 150% to 175% increase at 0.15 mM to 0.6 mM, while over 1.2 mM, a dose-dependent trend towards lower IFN-γ levels was observed (Figure 1B). Focusing specifically on the effect of 0.6 mM 2DG (the highest concentration achievable in vivo), we found that 0.6 mM 2DG significantly increased IFN-γ levels versus the untreated control (*p* < 0.05 as determined by Mann-Whitney test). This was further confirmed with a longer 72 h 2DG treatment in pre-activated T cells, where an even stronger stimulatory effect of 0.6 mM 2DG was observed with a 200% increase (*p* < 0.05) in IFN-γ levels (Figure 1C, Supplementary Figure S1B).

On the other hand, IL-2 levels exhibited a consistent dose-dependent decrease with 2DG treatment (Figure 1D). In contrast, CD69 expression was increased in a dose-dependent manner by 2DG in both T cell populations (Figure 1E,F, ns in CD8+ cells). PD-1 expression level was reduced by both 2DG concentrations to a similar degree (60% of control) in CD4+ cells, while in CD8+ cells, only a weak trend of decreased PD-1 (ns) was observed (Figure 1G,H). By analyzing PD-1 and CD69 positive and negative populations, we observed that 0.6 mM 2DG mainly increased the percentage of CD69+ PD-1- population in CD4+ cells, mostly at the expense of CD69- PD-1+ population (Figure 1I,J). In CD8+ cells, both CD69+ PD-1- and CD69+ PD-1+ populations were increased, while CD69- PD-1+ population was decreased (Figure 1K,L). Taken together, treatment with low 2DG concentration in pre-activated T-cells resulted in increased IFN-γ secretion and CD69 expression, reduced PD-1 expression in CD4+ cells, while higher 2DG concentration reduced IFN-γ secretion and strongly reduced IL-2 secretion without further reducing PD-1 levels (Supplementary Figure S1).

### 2.2. 2-Deoxy-D-Glucose Treatment Concurrent with Activation Does Not Increase IFN-γ Secretion in T Cells from PBMC

**Figure 2:**
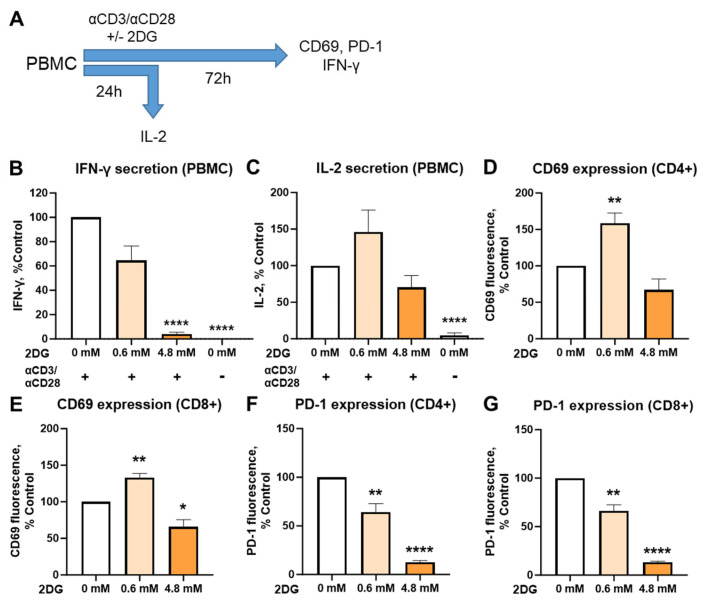
The effect of concurrent 2DG treatment on cytokine secretion, T cell activation and PD–1 expression in activated T cells from PBMC. PBMC were activated with anti-CD3 and anti-CD28 antibodies and treated for 72 h (24 h for IL-2 secretion) with 0.6 mM or 4.8 mM 2DG (denoted by shades of orange) during the activation (**A**). After treatment, supernatants were collected. The concentration of IFN-γ (**B**) and IL-2 (**C**) in supernatants were determined with enzyme-linked immunosorbent assay (ELISA). The cells were stained with antibodies and analyzed by flow cytometry. The expression levels of activation marker CD69 (**D**,**E**) and exhaustion marker/immune checkpoint PD-1 (**F**,**G**) were determined in CD4+ (**D**,**F**) and CD8+ (**E**,**G**) T cells. The raw data and gating strategy are shown in Supplementary Figure S2. The data represent the mean ± standard error of the mean (SEM) of six (**B**) or seven (**C**–**G**) independent experiments with different healthy donors. * *p* < 0.05, ** *p* < 0.01, **** *p* < 0.0001 as determined by repeated measures one-way analysis of variance (ANOVA) with Dunnett’s post-hoc test.

Next, we explored if the stimulatory effect of low concentration 2DG on IFN-γ secretion is present also when 2DG is present during the activation. We therefore activated the T cells from PBMC with anti-CD3/anti-CD28 antibodies in the presence or absence of low (0.6 mM) or high (4.8 mM) concentration of 2DG for 72 h (Figure 2A, Supplementary Figure S2A). We found about a 40% reduction in IFN-γ secretion with 0.6 mM 2DG (ns) compared to control, while 4.8 mM almost completely blocked IFN-γ secretion (Figure 2B). 4.8 mM 2DG treatment also led to slightly lower IL-2 levels (Figure 2C, ns) and CD69 expression (Figure 2D,E, ns in CD4+ cells). Interestingly, an opposite trend was observed for 0.6 mM 2DG, with IL-2 levels trending higher (ns) and CD69 expression being significantly increased in both CD4+ and CD8+ T cells. Surface PD-1 expression was decreased by 2DG in a dose-dependent manner in both CD4+ (Figure 2F) and CD8+ (Figure 2G) T cells, with 0.6 mM 2DG already decreasing the PD-1 levels to about 2/3 of control level. Overall, 0.6 mM 2DG treatment concurrent with activation did not lead to increased IFN-γ secretion. On the other hand, 2DG treatment concurrent with activation did reduce PD-1 expression while maintaining or increasing CD69 expression and IL-2 secretion in T cells from PBMC (Supplementary Figure S2).

### 2.3. 2-Deoxy-D-Glucose Reduces PD-1 Expression in Activated Jurkat Cells

**Figure 3:**
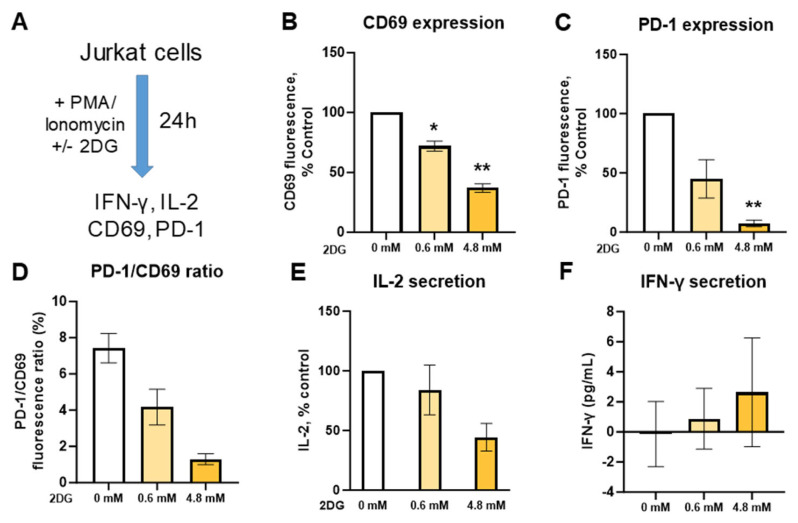
The effect of concurrent 2-deoxy-D-glucose treatment on IFN-γ secretion and activation markers in activated Jurkat cells. Jurkat cells were activated with PMA/ionomycin and treated with 0.6 mM or 4.8 mM 2DG as indicated for 24 h. (**A**) An overview of the treatment and activation protocol. (**B**–**D**) Relative surface expression of CD69 (**B**,**D**) and PD-1 (**C**,**D**) was determined by flow cytometry. The ratio of PD-1 and CD69 fluorescence is displayed in (**D**). (**E**,**F**) The concentration of IL-2 (**E**) and IFN-γ (**F**) in the medium was determined by ELISA. The mean ± SEM is shown for three independent experiments. * *p* < 0.05, ** *p* < 0.01, as determined by repeated measures one-way ANOVA with Dunnett’s post hoc test.

We then investigated the effect of low (0.6 mM) versus high (4.8 mM) 2DG concentration in activated Jurkat cells as another T cell model (Figure 3). We found that 2DG reduced the expression of the activation marker CD69 in a dose-dependent manner (Figure 3B). The same trend was observed for PD-1 expression (Figure 3C). The ratio of PD-1 to CD69 fluorescence was also decreased in a dose-dependent manner (Figure 3D, ns), suggesting a stronger effect on PD-1 expression versus activation. Although not significant, this effect was already observed at 0.6 mM 2DG, where the activation was only reduced by ~25%, suggesting a potential net beneficial effect on T cell responses. While 0.6 mM 2DG did not substantially reduce IL-2 secretion, there was a clear trend towards lower IL-2 levels with the 4.8 mM 2DG treatment (Figure 3E, ns). On the other hand, activated Jurkat cells secreted practically no IFN-γ, and 2DG treatment did not markedly affect this (Figure 3F). However, another clone of Jurkat cells not obtained directly from American Type Culture Collection (ATCC) did secrete more IFN-γ and exhibited a trend towards higher IFN-γ secretion with 2DG treatment while the effect of 2DG on CD69 and PD-1 expression was qualitatively similar (Supplementary Figure S3). Overall, 2DG reduced Jurkat cell activation and IL-2 secretion in a dose-dependent manner with a relatively weak effect at 0.6 mM concentration that already led to a substantial reduction in PD-1 levels, suggesting a potential net beneficial effect on T cell responses at low 2DG concentration.

### 2.4. The Stimulatory Effect of 2-Deoxy-D-Glucose on IFN-γ Secretion Is Partially Mediated by Inhibition of Protein N-Glycosylation

**Figure 4:**
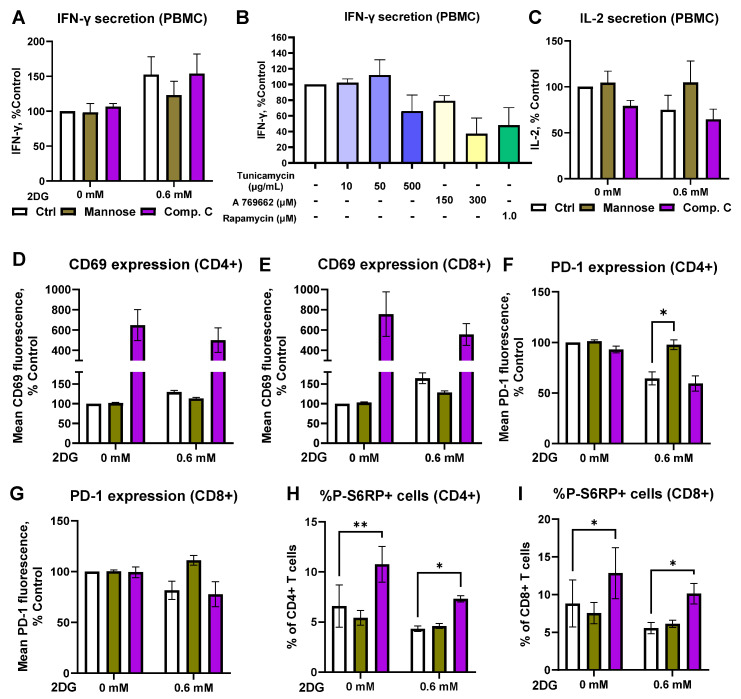
The role of protein N-glycosylation and AMPK activation in the effect of 2DG. PBMCs were activated with anti-CD3 and anti-CD28 antibodies for 48 h, harvested and washed, then treated for 24 h with 0.6 mM 2DG in the presence or absence of 2 mM mannose or 5 μM compound C (following 30 min pretreatment in case of the latter) ((**A**,**C**–**I**) Alternatively, the cells were treated after activation with tunicamycin, A 769662 or rapamycin as indicated (**B**). For cytokine secretion, the cells were restimulated with PMA and ionomycin for 4 h, after which cell culture supernatants were collected and the concentration of IFN-γ (**A**,**B**) and IL-2 (**C**) determined with ELISA. The expression levels of activation marker CD69 (**D**,**E**) and exhaustion marker/immune checkpoint PD-1 (**F**,**G**) were determined in CD4+ (**D**,**F**) and CD8+ (**E**,**G**) T cells using flow cytometry. The phosphorylation of S6RP in CD4+ (**H**) and CD8+ (**I**) T cells was determined using intracellular staining flow cytometry. The gating strategy and raw data are shown in Supplementary Figure S6. The data represent the mean ± SEM of three (**D**–**I**) to four (**A**–**C**) independent experiments with different healthy donors. * *p* < 0.05, ** *p* < 0.01 as determined by repeated measures one-way ANOVA (**B**) or repeated measures two-way ANOVA (**A**,**C**–**I**) with Dunnett’s or Šidak’s post hoc test.

The observed hormetic effect of 2DG on IFN-γ secretion led us to hypothesize that the mechanism responsible might involve the inhibition of protein N-glycosylation rather than inhibition of glycolysis, since 2DG already functions as a glycosylation inhibitor at low concentrations [14,16,17]. To test this hypothesis, we pre-activated T cells from PBMCs with anti-CD3/anti-CD28 antibodies for 48 h and then treated them with 0.6 mM 2DG in the presence of a low concentration of exogenous mannose to restore protein N-glycosylation. We found that mannose partially prevented the increase in IFN-γ secretion by 2DG (Figure 4A, ns) and the decrease in IL-2 secretion (Figure 4C, ns). Mannose also completely prevented the increase in IFN-γ secretion by 2DG in the Jurkat clone that secreted IFN—γ (Supplementary Figure S4). On the other hand, the protein N-glycosylation inhibitor tunicamycin did not show an increase in IFN-γ levels (Figure 4B) and showed a weaker effect on PD-1 levels than 2DG (Supplementary Figure S5). While the presence of mannose itself did not affect CD69 or PD-1 expression, mannose blocked the decrease in PD-1 expression that we have observed with 0.6 mM 2DG treatment (Figure 4D–G, significant only in CD4+ cells). Overall, the results suggest that the beneficial immunomodulatory effects of low concentration 2DG are indeed at least partially mediated by the inhibition of protein N-glycosylation.

As 2DG can also activate AMPK, we then investigated the role AMPK activation plays in the immunomodulatory effect of 2DG by pretreating the activated T cells from PBMCs with the AMPK inhibitor compound C. We found that compound C did not prevent the increase in IFN-γ secretion by 2DG (Figure 4A, ns). Conversely, the treatment with AMPK activator A 769662 led to unchanged or decreased IFN-γ secretion (Figure 4C, ns). Compound C also did not block the effect of 2DG on PD-1 expression (Figure 4F,G), but, interestingly, did lead to a substantial increase in CD69 expression on its own (Figure 4D,E, ns). Taken together, the results indicate that AMPK activation is not responsible for the effect of low-dose 2DG on IFN-γ secretion and PD-1 expression.

We then investigated whether 2DG could affect the mammalian target of the rapamycin (mTOR) pathway. We found no major effect of the 0.6 mM 2DG treatment on the phosphorylation of ribosomal protein S6 (S6RP, downstream mTORC1 target) with a weak trend towards lower P-S6RP levels (Figure 4H,I, ns). This was unaffected by the concurrent mannose treatment or compound C pretreatment, although the latter itself increased S6RP phosphorylation. Conversely, the mTORC1 inhibitor rapamycin slightly reduced IFN-γ secretion (Figure 4B, ns). The results therefore indicate that low-dose 2DG likely does not exert its effect on IFN-γ secretion via the mTOR pathway.

### 2.5. Low-Dose 2-Deoxy-D-Glucose Induces a Shift of Metabolism toward Increased Mitochondrial Resipiration Which Compensates Partially Inhibited Glycolysis

**Figure 5:**
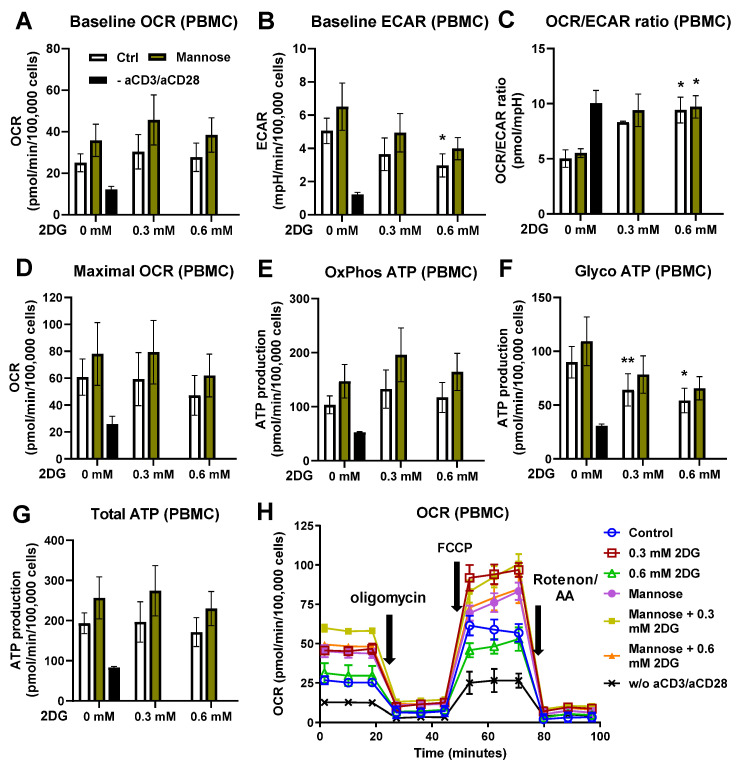
The effect of 2DG and mannose on energy metabolism of T cells from PBMCs. PBMCs were activated with anti-CD3 and anti-CD28 antibodies for 48 h, harvested and washed, then treated for 24 h with 0.3 mM or 0.6 mM 2DG in the presence or absence of 2 mM mannose. After treatment, the cells were harvested, counted, spun down and resuspended in Seahorse XF media without drugs, then seeded on Seahorse XFe24 cell culture plates. Baseline OCR (**A**), ECAR (**B**) and maximal OCR (**D**) were determined using Seahorse Mito Stress Test assay with 1.5 μM oligomicin, 2 μM carbonyl cyanide-p-trifluoromethoxyphenylhydrazone (FCCP) and 0.5 μM rotenone/antimycin A injections. The OCR/ECAR ratio (**C**) was calculated from baseline OCR and ECAR. The ATP production from oxidative phosphorylation (**E**), glycolysis (**F**) and total ATP production (**G**) were calculated according to the manufacturer’s instructions for Seahorse Real-Time ATP Assay. Mean ± SEM is shown for three independent experiments with different donors. * *p* < 0.05, ** *p* < 0.01 as determined by repeated measures of two-way ANOVA with Dunnett’s post hoc test. A representative time-course for OCR is shown in (**H**).

To further explore whether the modulatory effect of 2DG on IFN-γ secretion is associated with its direct effect on energy metabolism, we then analyzed the effect of low-dose (0.3 mM and 0.6 mM) 2DG on the glycolysis and oxidative phosphorylation in pre-activated T cells from PBMCs using the Seahorse real-time extracellular flux analysis. We found that 2DG lowered the extracellular acidification rate (ECAR) levels to approximately 60% (Figure 5A, *p* < 0.05 for 0.6 mM) with no major increase in the oxygen consumption rate (OCR) (Figure 5B, ns). Consequently, the OCR/ECAR ratio was significantly increased by 0.6 mM 2DG treatment (Figure 5C). Similarly, the ratio between the oxidative ATP produced and ATP produced from glycolysis (OxPhos/Glyco ATP) was increased by more than 2-fold (Figure S7A) by 0.6 mM 2DG, with the ATP production from glycolysis being significantly decreased (Figure 5F), while OxPhosATP was unaltered. Importantly, the total ATP production (Figure 5G) was not decreased by 2DG, and the maximal respiratory capacity (Figure 5C) was not affected. The shift in metabolism was essentially unaffected by the concurrent mannose treatment. While mannose did appear to especially boost the oxidative ATP generation (Figure 5A,E, ns), it did not prevent the increase in the OCR/ECAR ratio or the ratio of oxidative to glycolytic ATP production by 2DG (Figure 5C, Supplementary Figure S7). Overall, the results indicate a shift from glycolytic to oxidative metabolism with low-dose 2DG treatment, which does not impact the total energy-generating capacity of pre-activated T cells and is insensitive to mannose.

### 2.6. Low-Dose 2-Deoxy-D-Glucose Does Not Increase the Expression of Transcription Factors T-Bet and Eomesodermin

**Figure 6:**
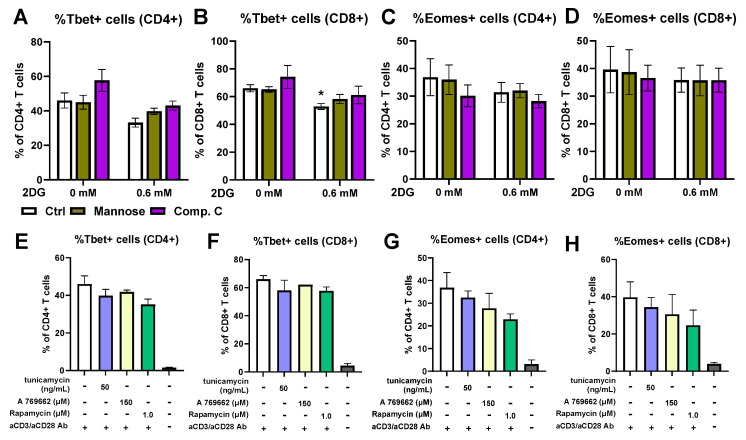
The effect of 2DG on transcription factors T-bet and Eomes. PBMCs were activated with anti-CD3 and anti-CD28 antibodies for 48 h, harvested and washed, then treated for 24 h with 0.6 mM 2DG in the presence or absence of 2 mM mannose (**A**–**D**). Alternatively, the cells were treated after activation with tunicamycin, A 769662 or rapamycin as indicated (**E**–**H**). The percentage of T-bet+ (**A**,**B**,**E**,**F**), Eomes+ (**C**,**D**,**G**,**H**), CD4+ (**A**,**C**,**E**,**G**) and CD8+ (**B**,**D**,**F**,**H**) T cells were determined using intracellular staining flow cytometry. The gating strategy is shown in Supplementary Figure S8. The data represent the mean ± SEM of three independent experiments with different healthy donors. * *p* < 0.05 as determined by repeated measures of two-way ANOVA (**A**–**D**) with Dunnett’s or Šidak’s post hoc test.

To investigate a possible mechanism of how a low-dose 2DG treatment leads to increased IFN-γ secretion, we measured the expression of the T-box transcription factor 21 (T-bet) and Eomesodermin (Eomes), two key transcription factors for IFN-γ expression [59,60]. We observed a slight trend towards a lower percentage of T-bet+ cells in both CD4+ (Figure 6A, ns) and CD8+ (Figure 6B) 2DG-treated T cells. This trend was partially reversed by the presence of mannose, but not compound C pretreatment. On the other hand, there was no clear effect of 2DG on Eomes expression (Figure 6C,D) regardless of mannose or compound C co-treatment. The percentage of both T-bet+ T cells (Figure 6E,F) and Eomes+ T cells (Figure 6G,H) was also not significantly altered by tunicamycin, AMPK activator A 679662 or rapamycin. Taken together, 2DG did not significantly increase the percentage of Eomes+ T cells and even slightly reduced the percentage of T-bet+ T cells.

### 2.7. Transcriptome Analysis Confirms a Dose-Dependent Induction of Unfolded Protein Response and the Absence of Increased IFNG Transcription with 2DG Treatment

**Figure 7:**
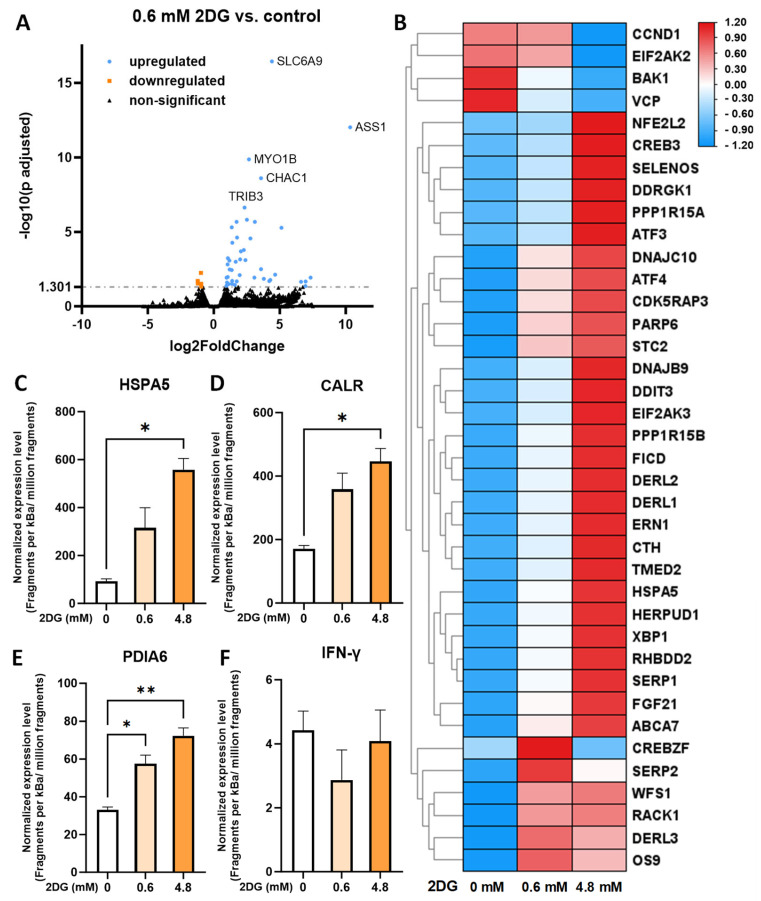
The transcriptomics analysis of 2DG-treated T cells from PBMCs. PBMCs were activated with anti-CD3 and anti-CD28 antibodies for 48 h, harvested and washed, then treated with 0.6 mM or 4.8 mM 2DG for 24 h. After treatment, the cells were harvested, washed and snap frozen in liquid nitrogen. Total mRNA was isolated and the relative expression levels of individual mRNA determined with RNAseq. (**A**) The volcano plot of differentially expressed genes in 0.6 mM 2DG-treated versus control cells with top five most significantly altered genes marked. (**B**) Heatmap analysis of genes involved in the unfolded protein response (UPR). The z-score displayed on the heatmap was calculated from FPKM. (**C**–**F**) The relative mRNA expression levels of chaperones HSPA5 (**C**), calreticulin (**D**), PDIA6 (**E**) and IFN-γ (**F**). The data represent the mean ± SEM of three independent experiments with different healthy donors. * *p* < 0.05, ** *p* < 0.01 as determined by repeated measures of one-way ANOVA (**C**–**F**) with Dunnett’s post hoc test.

To investigate the effect of 2DG treatment on RNA transcription in pre-activated T cells, we performed a transcriptome analysis using RNA sequencing. We found that the 0.6 mM 2DG treatment induced the upregulation of forty-three genes, including *SLC6A9*, *ASS1*, *MYO1B* and *CHAC1*, while there were only five downregulated genes (Figure 7A). The transcriptional changes induced by 4.8 mM 2DG were even more pronounced with 785 upregulated and 963 downregulated genes (Supplementary Figure S9A). The 2DG treatment induced markers of ER stress (Supplementary Figure S9B) and the subsequent unfolded protein response (UPR, Figure 7B) in a dose-dependent manner. With the exception of *CREBZF*, *SERP2* and *TMEM259*, whose expression was highest with 0.6 mM 2DG (ns versus control), the mRNA levels of involved genes in 0.6 mM 2DG-treated cells were intermediate between control and 4.8 mM 2DG. The same pattern held true for the *ATF4* and *XBP1*, two of the main markers of UPR (Supplementary Figure S9), and for the individual chaperones *HSPA5*, *CALR*, *PDIA6*, *PDIA4* and *GRP94* (*HSP90B1*) (Figure 7C–E, Supplementary Figure S9) involved in IFN-γ folding in the ER [61]. Among these chaperones, only PDIA6 was significantly increased by 0.6 mM 2DG as assessed by RM ANOVA (Figure 7), while HSPA5 (-logP 4.6), CALR (-logP 1.9) and PDIA4 (-logP 1.5) were significantly increased by 0.6 mM 2DG versus control according to volcano plot analysis. Interestingly, *IFNG* was not significantly increased by the 2DG treatment regardless of the concentration (Figure 7F). Nevertheless, there was a slight tendency for an upregulation of interferon-stimulated genes (ISGs), such as *STAT1*, *IRF9*, *GBP5* and *CD274* (*PD-L1*), when exposed to 0.6 mM 2DG (Supplementary Figure S11). Taken together, the transcriptome analysis confirmed the dose-dependent induction of ER stress and UPR by the 2DG treatment with no increase in *IFNG* transcription, but rather an upregulation of the interferon-stimulated genes.

## 3. Discussion

2-deoxy-D-glucose (2DG) is a promising cancer treatment adjuvant, particularly in the context of cancer immunotherapies due to its indirect immunomodulatory effects via immunogenic tumor cell death [34] and reduced PD-L1 and PD-1 glycosylation and surface expression [38,39,40,41]. Additionally, 2DG treatment of T cells ex vivo can improve their proliferative capacity and treatment outcomes in the context of adoptive cell therapies such as CAR T [42,56,57]. On the other hand, 2DG as a glycolysis inhibitor can reduce the levels of IFN-γ, an important effector cytokine for the anti-tumor immune response, in both rodent models [53,54,55] and in vitro studies on human T cells from PBMCs [46,50,51,52] in line with the crucial role of glycolysis in IFN-γ secretion [46,48,49]. However, a recent study found that the two-week 2DG treatment induced a NK-like T cell phenotype associated with increased cytotoxicity and the secretion of key effector cytokines including IFN-γ [57]. It is therefore crucial to establish which factors determine the effect of 2DG on T cells. The concentration of 2DG can strongly influence its effect, particularly since the inhibition of glycolysis requires higher concentrations than the inhibition of protein N-glycosylation. Previous in vitro studies of 2DG on human T cells from PBMCs often used high (≥2 mM) concentrations to block glycolysis [46,52]. While this is highly relevant in the context of adoptive therapy where the treatment takes place ex vivo [56,57], it is not directly applicable to the use of 2DG as an adjuvant therapy, since the concentration of 2DG safely achievable in the plasma of human patients is around 0.6 mM [32,58]. We therefore investigated whether low, physiologically achievable 2DG concentrations (up to 0.6 mM) could also have a stimulatory effect on IFN-γ secretion, which could be beneficial as an adjuvant for immune checkpoint therapy.

Since 2DG can directly affect T cell activation [40,50,62], it is important to consider the timing of 2DG treatment in relation to T cell activation. To that end, we investigated both the effect of 2DG treatment concurrent as T cell activation with anti-CD3/anti-CD28 antibodies, as well as the effect of 2DG treatment of T cells pre-activated 48 h prior in the absence of 2DG. Our results showed a marked difference in the effect of 2DG between the two protocols. On the one hand, when T cells were first activated with anti-CD3/anti-CD28 antibodies for 48 h and only then treated with 2DG for 24 h followed by 4 h restimulation with PMA and ionomycin, low concentrations of 2DG up to 0.6 mM increased the IFN-γ secretion (Figure 1). On the other hand, the concurrent 2DG treatment of T cells from the start of activation resulted in decreased IFN-γ secretion regardless of 2DG concentration (Figure 2), consistent with previous studies [50,51,52]. The different timing of the treatment, together with different 2DG concentration, could therefore explain the existing discrepancy regarding the stimulative versus inhibitory effect of 2DG on T cell immunity in existing literature. Mechanistically, the different results observed between the two protocols could be explained with the inhibitory effect of 2DG on the T cell activation—when 2DG is administered at the beginning of activation it prevents full activation of T cells. Renner et al. showed that 2DG, as opposed to glucose withdrawal, can block the increase in oxidative metabolism involved in early T cell activation, preventing full activation and the engagement of effector functions [50]. In terms of metabolic phenotype, we observed that a partial shift from glycolytic to mitochondrial metabolism at 0.6 mM 2DG with no increase in total ATP when T cells were treated after activation. Together with no effect on mTOR this can explain why a glycolysis inhibitor 2DG can even increase IFN-γ and CD69.

However, we should note that 0.6 mM 2DG consistently increased CD69 levels regardless of treatment timing and even showed a trend towards higher IL-2 levels when administered at the beginning of activation. Therefore, it is possible that restimulation with PMA and ionomycin also plays a role in determining the response of T cells to 2DG, since the observed increase in IFN-γ levels was also observed in the PMA/ionomycin stimulated Jurkat cell clone not directly obtained from ATCC (Supplementary Figure S3). However, the short (4 h) PMA/ionomycin restimulation protocol is well established in T cell immunology to investigate the secretion of cytokines (particularly for intracellular cytokine staining). Another thing to note is the potential effect of other cell types present in PBMC, such as B cells, natural killer cells and monocytes, which could potentially also contribute to IFN-γ secretion, despite the large T cell fraction (≥70%) after activation with CD3/CD28 antibodies (Supplementary Figure S12A) and monocyte removal by adhesion to cell culture plates. To confirm that the major effect on IFN-γ is due to T cells, we have performed additional experiment in isolated T cells from PBMC (Supplementary Figure S12B). In agreement with our previous results, we obtained a similar stimulatory effect of 0.6 mM 2DG in isolated T cells on IFN-γ secretion, confirming that stimulation of T cells contributed to the observed effects of 2DG in pre-activated T lymphocytes.

Interestingly, the low-dose 2DG treatment affected the IFN-γ signaling pathway even without restimulation. We observed slightly increased mRNA levels of *STAT1*, a pivotal transcription factor activated by phosphorylation in response to IFN-γ signaling [63] with 0.6 mM 2DG, while 4.8 mM 2DG decreased it. *STAT1* mRNA levels thus showed a similar trend to IFN-γ secretion, in agreement with a previous study showing that 2DG in higher concentration reduced STAT1 phosphorylation in IFN-γ activated human peripheral blood monocytes [64]. Furthermore, STAT1 activation by the IFN-γ receptor complex is responsible for the transcription of a significant portion of ISGs, including *IRF9*, *GBP5* and *CD274*, which were also slightly upregulated with 0.6 mM 2DG [65]. These results thus suggest that low-dose 2DG treatment increases IFN-γ signaling in PBMCs.

The stimulatory effect of low-dose 2DG on IFN-γ was specific to this cytokine and not cytokine secretion in general, since 2DG treatment of pre-activated T cells led to a consistent dose-dependent drop in IL-2 levels. In contrast, IFN-γ levels exhibited a clear hormesis, increasing IFN-γ secretion for low 2DG concentrations up to 0.6 mM, while higher concentrations led to reduced IFN-γ levels. This could not be fully explained by reduced T cell proliferation or viability, since even with 4.8 mM 2DG, the cells were ≥90% viable and no further drop in cell number from 0.6 mM 2DG was observed (Figure S1). Instead, the reduced IFN-γ secretion at high concentrations can be explained by the inhibition of glycolysis, which is required for both the IFN-γ transcription and translation [48,49]. However, since there was already a small significant decrease in glycolysis with compensatory increase in mitochondrial oxidative metabolism at 0.6 mM 2DG (Figure 5), another mechanism must be responsible for the increase in IFN-γ at low 2DG concentration.

2DG is a structural analog of mannose and can compete with it for incorporation into glycans, thus disrupting protein N-glycosylation [14,15,16,17]. This effect was responsible for the increased cytotoxicity and IFN-γ secretion in T cells treated with 2 mM 2DG for two weeks, as the effect of 2DG was prevented by exogenous mannose [57]. In our study, mannose only very weakly abrogated (ns) the increased IFN-γ secretion by short-term 0.6 mM 2DG treatment (Figure 4), despite not preventing the partial shift from glycolysis to oxidative metabolism with 2DG. However, mannose prevented the 2DG-induced increase in IFN-γ secretion in the Jurkat clone that secreted more IFN-γ (Supplementary Figure S4). This indicates that while altered protein N-glycosylation likely plays a role in the increased IFN-γ secretion in Jurkat cells, we could not confirm this in primary T cells. Additionally, tunicamycin, another protein N-glycosylation inhibitor [66,67], did not recapitulate the effect of low-dose 2DG on IFN-γ in PBMCs (Figure 4B), though a weak trend towards higher levels was observed in the Jurkat clone (Supplementary Figure S4). This is consistent with the role of N-glycosylation for IFN-γ secretion and stability, as the complete prevention of N-glycosylation (e.g., by tunicamycin) can reduce its secretion [68,69]. In contrast, 2DG treatment alters the N-glycan structure but does not prevent the N-glycan assembly or transfer altogether [57]. Taken together, these results indicate that, while we cannot completely exclude the role of altered protein N-glycosylation in the increased secretion of IFN-γ by short-term 2DG treatment in human T cells, it is not itself sufficient for this effect.

The mechanism by which the 2DG treatment leads to increased IFN-γ secretion is not yet entirely clear. Sasawatari et al. have demonstrated that two-week 2DG treatment increased IL-2R retention on the cell surface, leading to greater IL-2 signaling by exogenous IL-2 [57]. Additionally, 2DG increased the expression of Eomes, a key transcription factor for IFN-γ transcription in CD8+ T cells [57,60]. However, in our study, no exogenous IL-2 was administered and there was no observable increase in either Eomes or T-bet expression (Figure 6) or in *IFNG* transcription (Figure 7). All these data indicate that there is likely another separate mechanism involved in the increased IFN-γ secretion by 2DG treatment that is already involved in short-term treatment.

We speculated that ER stress and the subsequent activation of the unfolded protein response (UPR), clearly demonstrated for 2DG in other cell types [16,18,19,22], could be responsible for this effect. Our results show that both markers of ER stress and UPR were induced by 2DG treatment in PBMCs at the mRNA level in a dose-dependent manner (Figure 7, Supplementary Figure S9). It is known that ER stress and UPR can lead to the increased secretion of other pro-inflammatory cytokines from monocytes or dendritic cells [70,71]. Additionally, UPR components play a vital role in T cell function. For example, ATF4 is required for the Th1 differentiation of mouse CD4+ T cells [72]. Additionally, naïve T cells activation causes the integrated stress response with eIF2α phosphorylation via several pathways including the UPR kinase PERK. This results in the accumulation of transcribed mRNAs of effector cytokines, including IFN-γ, that are translated and secreted only after TCR restimulation [73]. However, it is presently unclear if the induction of ER stress by exogenous stimuli could increase IFN-γ levels. It is established that chronic ER stress can reduce T cell function by inhibiting the oxidative metabolism in T cells via XBP1 [74] or decreasing T-bet expression via PERK, ATF4 and Chop in tumor-infiltrating CD8+ T cells [75]. It is therefore important to note that, in our experiments, the induction of ER stress was short-term and not chronic. Much less is known about acute ER stress and IFN-γ secretion and, to the best of our knowledge, our study is the first to suggest that short-term, intermediate-level ER stress could lead to increased IFN-γ secretion in the context of 2DG treatment.

Several mechanisms could theoretically link ER stress and/or the subsequent UPR to increased IFN-γ secretion, including the potentiated priming of *IFNG* mRNA prior to eIF2α de-phosphorylation as discussed above. Alternatively, increased Ca^2+^ leakage from the ER caused by ER stress could lead to higher cytosolic Ca^2+^ levels [76] and NFAT1 activation [77], increasing the transcription of *IFNG* [78] together with T-bet. However, we observed no increase in Eomes expression. The percentage of T-bet+ cells was even slightly reduced, consistent with the effect of chronic ER stress [75] even though the 2DG exposure was short-term (24 h). Altered Ca^2+^ signaling should alter both IFN-γ and IL-2 levels in accordance with Ca^2+^ channel studies [79,80], but this was not observed as neither *NFATC1* and *NFATC2* (Supplementary Figure S10) nor *IFNG* levels were increased by low 2DG. Altogether, this indicates that the increase in IFN-γ secretion is probably not caused by enhanced transcription. The mechanism of increased IFN-γ secretion with short-term 2DG therefore appears to involve changes downstream of transcription. A substantial increase in *IFNG* translation appears unlikely without an increase in mTORC1 activity (Figure 4H–I). We were also unable to find any published evidence for the preferential translation of IFN-γ during ER stress. However, IFN-γ is glycosylated [81] and folded by multi-chaperone complexes in the ER prior to secretion [61], and the expression of chaperones involved in IFN-γ folding (*HSPA5*, *CALR*, *PDIA6* and *PDIA4*) were upregulated or showed a trend towards upregulation by 0.6 mM 2DG treatment at the mRNA level (Figure 7, Supplementary Figure S9). Since ER chaperones are also preferentially translated during ER stress, this could increase the ER folding capacity for the eventual secretion of IFN-γ during restimulation.

It is important to note that 2DG can also activate AMPK both via the energy stress induced by glycolysis inhibition and increased cytosolic Ca^2+^, as discussed above, and subsequent CaMKKβ activation [19]. Though the latter mechanism takes place after T cell activation via the TCR [82], AMPK activation can also restrict the expression of IFN-γ in T cells [83,84]. This is consistent with our results using the direct AMPK activator A 769662 (Figure 4B). Conversely, the AMPK inhibitor compound C showed a trend towards higher IFN-γ levels, while low-dose 2DG treatment in the presence of compound C led to the same or a greater increase in IFN-γ secretion. These results therefore suggest that AMPK activation restricts the IFN-γ secretion in T cells treated with higher concentrations of 2DG and could further help to explain the observed hormetic effect with the increase in IFN-γ only at low-dose 2DG.

It is very interesting to note that the concentration range at which 2DG can increase IFN-γ secretion with short-term treatment is also the concentration range achievable in vivo [32]. This means that the observed effect on IFN-γ secretion could potentially be relevant in the context of anti-tumor immunity in vivo. Even though the role of IFN-γ is complex [85], it can directly reduce cancer cell proliferation [86] and is one of the key effector cytokines in anti-tumor T cell responses. The stimulatory effect of low 2DG could therefore potentially improve the anti-tumor response. Some caution is still advised, since the effect of 2DG seems to be dependent on the timing of the 2DG treatment relative to T cell activation. Nevertheless, we should point out that low-dose 2DG reduced PD-1 expression (in agreement with [41]) and increased CD69 expression regardless of the timing of the treatment, which could also beneficially impact the anti-tumor T cell response by rendering the T cells less susceptible to inhibition via the PD-1/PD-L1 axis. The fact that IL-2 levels were also not substantially decreased by 0.6 mM 2DG also suggests at most a modest suppression of other effector functions. Overall, the results suggest that low-dose 2DG could potentially improve the anti-tumor T cell response not only by decreasing PD-L1 expression on tumor cells [38,39,40], but also by reducing PD-1 expression on T cells and boosting their secretion of IFN-γ.

## 4. Materials and Methods

### 4.1. Cell Culture, PBMC Isolation and Treatments

Jurkat cells were acquired from ATCC and maintained in ATCC-modified RPMI 1640 medium with 25 mM glucose, 2 mM glutamine and 1 mM pyruvate (Gibco, Thermo Fisher, Waltham, MA, USA) supplemented with 10% fetal bovine serum (FBS) (Sigma-Aldrich, Saint Louis, MO, USA). All experiments were performed in RPMI 1640 medium (Genaxxon bioscience GmbH, Ulm, Germany) supplemented with 10% FBS, 5.6 mM glucose and 2 mM glutamine (Sigma-Aldrich, Saint Louis, MO, USA) unless otherwise indicated. The cells were seeded at 5 × 10^5^/mL, activated with 25 ng/mL PMA and 1.0 µM ionomycin (both from Sigma-Aldrich, Saint Louis, MO, USA) for 24 h and treated with 0.6 mM or 4.8 mM 2DG at the same time as indicated.

Peripheral blood mononuclear cells (PBMCs) were isolated from buffy coats of healthy donors by Ficoll gradient centrifugation as previously described [62]. Briefly, peripheral blood was allowed to cool to room temperature for 30 min, diluted with 2 mM ethylenediaminetetraacetic acid (EDTA) in phosphate-buffered saline (PBS) and carefully placed on a layer of Ficoll-Paque^®^ Premium (GE Healthcare Bio-Sciences AB, Uppsala, Sweden). Separation was performed by centrifugation at 400× *g* for 40 min without active braking. The resulting buffy coats were aspirated and washed three times with 2 mM EDTA in 1 × PBS. PBMCs were suspended in complete RPMI medium (RPMI 1640 medium (Genaxxon bioscience GmbH, Ulm, Germany) supplemented with 10% autologous serum, 5.6 mM glucose and 2 mM glutamine), counted and diluted to 1 × 10^6^ cells/mL. Anti-CD28 antibody (302934, Biolegend, San Diego, CA, USA) was added to the final concentration of 5 μg/mL and the cell were seeded onto cell culture plates pre-coated with anti-CD3 antibodies (300438, Biolegend, San Diego, CA, USA). The plates were coated for 2 h at 37 °C using 300 µL/well for 24-well and 150 µL/well for 48-well plates of 10 μg/mL antibody in PBS and washed three times with 1 × PBS prior to PBMC seeding. In some experiments, 0.6 mL of cells were seeded on 48-well plates and treated with 0.6 mM and 4.8 mM 2DG during the activation for 72 h. In other experiments, 1.2 mL of cells per well were seeded on 24-well plates and activated for 48 h, after which the cells were harvested, spun down and resuspended in fresh medium without antibodies, and 0.6 mL of cells per well at 1 × 10^6^ cells/mL was seeded on 48-well plates. The cells were then treated with 2DG as indicated for 24 h, after which the analyses were performed unless otherwise indicated.

### 4.2. Cytokine Secretion

Jurkat cells were seeded on 12-well plates at 5 × 10^5^ cells/mL in complete RPMI with 5.6 mM glucose and treated with 0.6 mM or 4.8 mM 2DG in media with 25 ng/mL PMA and 1.0 µM ionomycin for 24 h as indicated. The cells were harvested by centrifugation and the supernatants collected and stored at −80 °C until further analysis. PBMCs were seeded and activated with anti-CD3/anti-CD28 antibodies as described above. In case of concurrent treatment and activation, the media samples were collected after 24 h (for IL-2) and 72 h (for IFN-γ) of activation. In experiments with 48 h activation followed by 24 h 2DG treatment RPMI with 5.6 mM glucose was used, for experiment with 72 h activation followed by 72 h treatment RPMI with 25 mM glucose was used. For experiment in isolated T cells from human PBMC, PBMC were washed following by isolation of T cells with the MojoSort Human CD4 T/CD8 T Cell Isolation kit (Biolegend, San Diego, CA, USA). Isolated T cells were seeded and activated with anti-CD3 and anti-CD28 antibodies for 72 h. The T cells were treated for 72 h with 0.3 mM or 0.6 mM 2DG. After the treatment period 25 ng/mL PMA and 1.0 µM ionomycin was added for 4 h, after which the media samples were collected. In all cases, the cells were harvested and spun down, and the supernatants were collected and stored at −80 °C until further analysis. The concentration of IL-2 and IFN-γ in supernatants was determined using human IL-2 ELISA kit (88-7025, Invitrogen, Waltham, MA, USA) and human IFN-γ ELISA kit (88-7316, Invitrogen, Waltham, MA, USA) according to the manufacturer’s instructions.

### 4.3. Cell Surface Markers and Transcription Factors Expression

Jurkat cells were seeded on 12-well plates at 5 × 10^5^ cells/mL in complete RPMI and treated with 0.6 mM or 4.8 mM 2DG in media with 25 ng/mL PMA and 1.0 µM ionomycin for 24 h as indicated. The cells were then harvested by centrifugation and stained with APC-conjugated anti-PD-1 (329908, Biolegend, San Diego, CA, USA) and Pacific Blue conjugated anti-CD69 (310920, Biolegend, San Diego, CA, USA) at room temperature for 20 min. Cells were then washed with PBS with 1% bovine serum albumin (BSA), resuspended in PBS and analyzed on Attune NxT flow cytometer (Thermo Fisher Scientific, Waltham, MA, USA).

PBMCs were isolated, cultured and treated as described above. After treatment, the cells were harvested and stained at room temperature for 20 min with the following antibodies (obtained from Biolegend, San Diego, CA, USA): APC/Cy7 conjugated anti-CD3 (300318), PerCP-Cy5.5 conjugated anti-CD4 (300530), FITC conjugated anti-CD8 (300906), APC-conjugated anti-PD-1 (329908) and Pacific Blue conjugated anti-CD69 (310920). Cells were then washed with PBS with 1% BSA, resuspended in PBS and analyzed on Attune NxT flow cytometer. For staining of intracellular transcription factors, the cells were washed after performing the surface staining protocol, then fixed and permeabilized using the True-Nuclear™ Transcription Factor Buffer Set (Biolegend, San Diego, CA, USA) according to manufacturer’s instructions. The following antibodies were used for intracellular staining according to manufacturer’s instructions: PE-conjugated anti-Eomes (566749, BD Biosciences, Franklin Lakes, NJ, USA), BV421™-conjugated anti-T-bet (644816, Biolegend, San Diego, CA, USA) and BV421™-conjugated anti-P-S6RP (608609, Biolegend, San Diego, CA, USA). After staining, the cells were washed, resuspended in PBS and analyzed on Attune NxT flow cytometer.

### 4.4. Real-Time Metabolic Assay

PBMCs were isolated and activated for 48 h with anti-CD3/anti-CD28 antibodies as described above. The cells were then washed and treated in antibody-free medium with 2DG and/or 2 mM mannose for 24 h as indicated. The cells were then harvested, counted, resuspended in Seahorse XF RPMI 1640-based Seahorse XF Glycolytic Rate Assay Medium (5.6 mM glucose, 2 mM L-glutamine, 0 mM sodium pyruvate, 1 mM HEPES, equilibrated to pH 7.4) (Agilent Technologies, Santa Clara, CA, USA) and plated on a 24-well Seahorse cell culture plates covered with CellTak^®^ at 2.75 × 10^5^ in 0.1 mL per well. The plates were spun down at 200 g for 1 min and incubated at 37 °C for 15 min without CO_2_. Seahorse XF medium (0.4 mL) was then added and the plate was incubated for an additional 30 min at 37 °C without CO2. The Seahorse Mito Stress Assay was performed using 1.5 μM oligomycin, 2 μM FCCP and 0.5 μM rotenone/antimycin A (Agilent).

The maximal respiration (maximal OCR) was determined according to Mito Stress Assay. Glycolytic ATP production was calculated as glycolytic proton efflux rate according to the following equation:

glycoATP Production Rate (pmol ATP/min) = glycoPER (pmol H+/min)

= basalPER (pmol H+/min) − MitoPER (pmol H+/min)

= basalPER − (basal OCR − OCR after rotenone/antimycin A) × 0.6.

The oxidative phosphorylation ATP production rate (OxPhosATP) was calculated according to the following formula:

OxPhosATP (pmol ATP/min) = (basal OCR − OCR after oligomycin)

(pmol O_2_/min) × 2 (pmol O/pmol O_2_) × P/O (pmol ATP/pmol O_2_)

Assuming a P/O ratio of 2.75, the total ATP production was defined as the sum of glycolytic and oxidative phosphorylation ATP production.

### 4.5. Transcriptome Sequencing

Activated PBMCs (48 h activation with aCD3/aCD28) were treated with 0.6 mM or 4.8 mM 2DG in RPMI media with 5.6 mM glucose. After 24 h, the cells were washed twice with ice-cold PBS, snap frozen in liquid nitrogen and stored at −80 °C. Total cell RNA was isolated from cell pellets using PureLink™ RNA Mini Kit (Invitrogen, ThermoFisher Scientific, Waltham, MA, USA). Transcriptome sequencing of PBMCs was performed by Novogene (Cambridge, UK) and the sequencing was performed in triplicate in each group. The values of fragments per kilobase of transcript per million mapped reads (FPKM) were used to compare gene’s expression level and to generate heatmaps, which were then visualized using TBtools (TBtools-II v2.119) [87].

### 4.6. Statistical Analysis

The statistical analysis was performed using GraphPad Prism (v9; GraphPad Software, Inc., La Jolla, CA, USA). The results were displayed as mean ± SEM unless indicated otherwise. For Jurkat cells, three to four independent experiments were performed. For PBMCs, three to four independent experiments with different individual donors were performed unless indicated otherwise. The statistical significance was tested using repeated measures one-way or two-way ANOVA with Dunnett’s or Šidak’s post hoc test. Unless indicated otherwise, *p*-value under 0.05 was considered statistically significant.

### 4.7. Ethics Approval Statement

The study was carried out in concordance with the Declaration of Helsinki and was approved by the National Medical Ethic Committee (approval number 0120-255/2021/3). The participants provided their written informed consent to participate in this study.

## 5. Conclusions

In the present study, we investigated the short-term effect of 2DG on human T cells from PBMCs. We found that low, physiologically achievable concentrations of 2DG up to 0.6 mM increased the secretion of IFN-γ, whereas higher concentrations over 2.4 mM had a suppressive effect. This effect was specific to IFN-γ versus IL-2, whose levels declined monotonously with 2DG concentration. The stimulatory effect of low 2DG on IFN-γ was only present when T cells were pre-activated with anti-CD3/anti-CD28 antibodies for 48 h and only then treated with 2DG, followed by restimulation with PMA and ionomycin. On the other hand, the 2DG treatment concurrent with activation led to a decrease in IFN-γ secretion even with 0.6 mM 2DG, likely as a result of partial suppression of the initial T cell activation.

The main mechanism for increased IFN-γ secretion with short-term 2DG treatment of pre-activated T cells appears not to involve changes in *IFNG* transcription or signaling upstream of it, but rather a process downstream of transcription. We propose the increased folding capacity of the ER due to induced expression of chaperones involved in IFN-γ folding as a possible mechanism linking the effects of 2DG (e.g., ER stress) to increased IFN-γ secretion. We further suggest that this effect is only observed at low 2DG concentrations due to AMPK activation (via energy stress due to glycolysis inhibition and/or strong ER stress and CaMKKβ) at higher 2DG concentrations restricting IFN-γ secretion, leading to the observed hormesis.

As the concentration range where 2DG can increase the IFN-γ secretion is achievable in vivo, low-dose 2DG as an adjuvant could potentially increase IFN-γ secretion in the context of anti-tumor immunity. The different results depending on the treatment timing warrant some caution and further investigation. Nevertheless, the reduction in PD-1 expression and increased CD69 expression, together with the lack of strong IL-2 suppression by the 0.6 mM 2DG concentration, suggest that a low concentration of 2DG could indeed have potentially beneficial effects on anti-tumor immunity regardless of the treatment timing.

## Data Availability

The original contributions presented in the study are included in the article/Supplementary Materials; further inquiries can be directed to the corresponding author.

## Supplementary Materials

The following supporting information can be downloaded at https://www.mdpi.com/article/10.3390/ijms251910384/s1.
