# Supplementary material for: Physiologically Achievable Concentration of 2-Deoxy-D-Glucose Stimulates IFN-γ Secretion in Activated T Cells In Vitro
Source: Int J Mol Sci. 2024 Sep 26;25(19):10384. doi: 10.3390/ijms251910384 (PMC11476708; doi:10.3390/ijms251910384)
Supplement: Supplementary file 1 [file ijms-25-10384-s001.zip › ijms-3149486-supplementary.pdf]

Supplementary information

Title: **Physiologically achievable concentration of 2-deoxy-D-glucose stimulates IFN-γ secretion in activated T cells in vitro**

Jernej Repas, Tjaša Frlic, Tadeja Snedec, Andreja Nataša Kopitar, Harald Sourij, Andrej Janež and Mojca Pavlin*

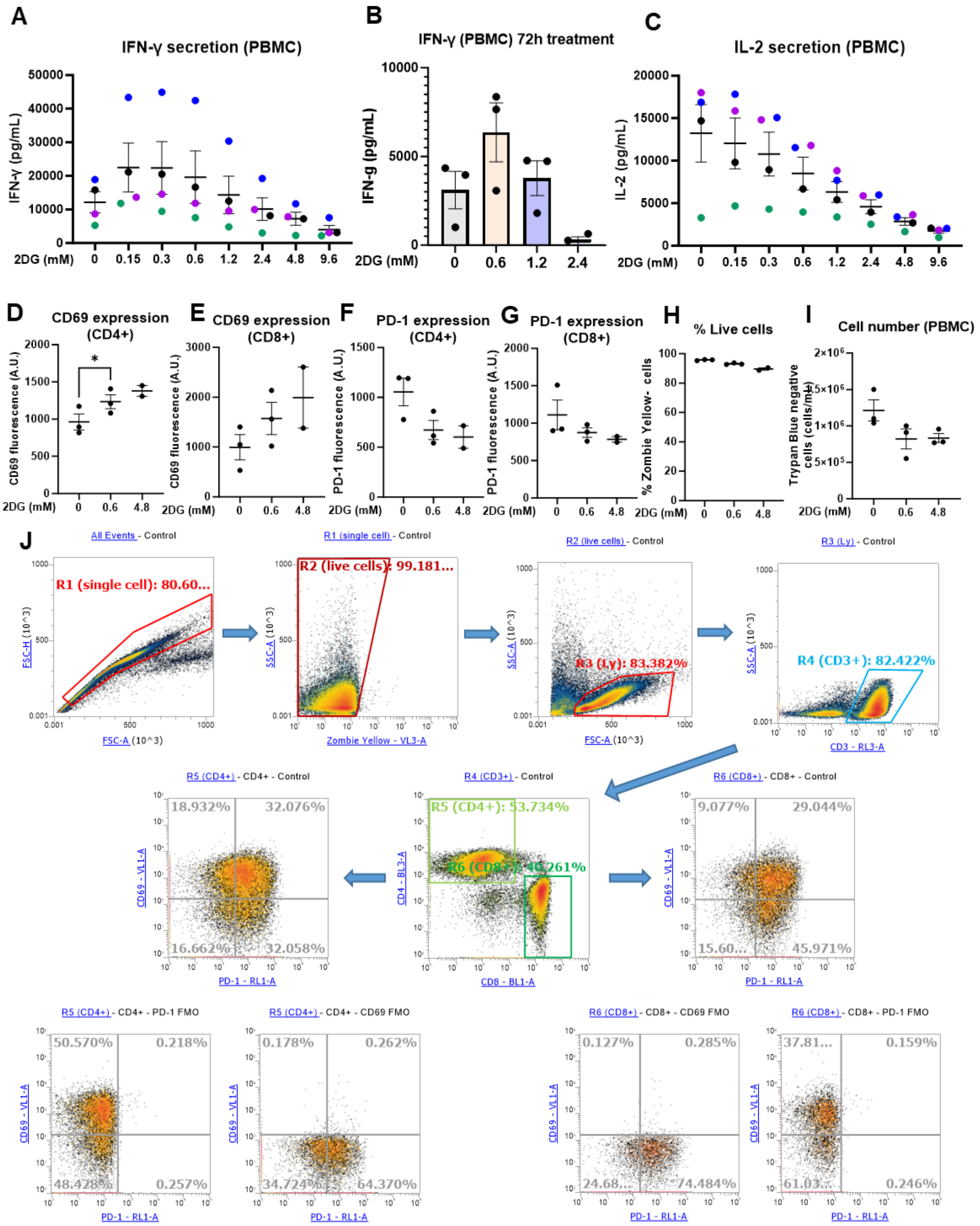

Figure S1: The effect of 2DG treatment on cytokine secretion, T cell activation and PD-1 expression in pre-activated T cells from PBMC. PBMC were activated with anti-CD3 and anti-CD28 antibodies for 48h (**A**, **B**) or 72h (B), harvested and washed, then treated for 24h with 0.6 mM or 4.8 mM 2DG (denoted by shades of orange) in Fig. **B** cell were treated for 72h, stained with antibodies and analyzed with flow cytometry. For cytokine secretion, the cells were restimulated after the treatment with PMA and ionomycin for 4h, after which media supernatants were collected. The concentration of IFN-γ (**A,B**) and IL-2 (**C**) in supernatants were determined with ELISA. The expression levels of activation marker CD69 (**D E**) and exhaustion marker/immune checkpoint PD-1 (**F**, **G**) were determined in CD4+ (**D**, **F**) and CD8+ (**E**, **G**) T cells using flow cytometry. The individual data points represent individual donors, while the horizontal lines represent the mean ± SEM**D-H**) to four (**A,C**) independent experiments with different healthy donors. *p<0.05 as determined by repeated measures one-way ANOVA with Dunnett's post-hoc test. (**H**) The percentage of live cells (Zombi Yellow -) and (**I**) the number of Trypan Blue negative cells is shown. (**J**) The gating strategy for the analysis of CD69 and PD-1 expression by flow cytometry. The gates for CD69+ and PD-1+ populations were set according to fluorescence minus one (FMO) controls.

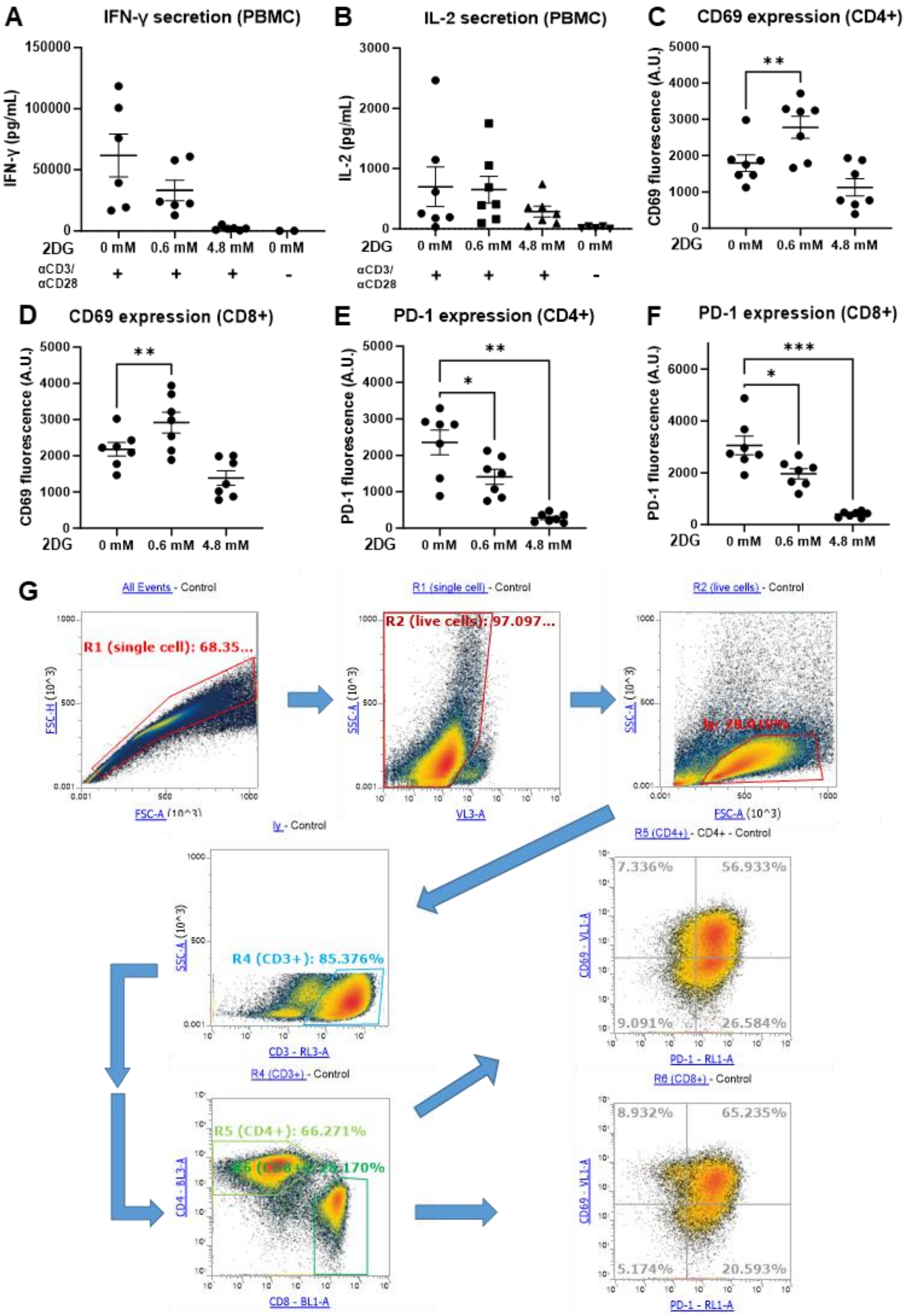

Figure S2: The effect of concurrent 2DG treatment on cytokine secretion, T cell activation and PD-1 expression in activated T cells from PBMC. PBMC were activated with anti-CD3 and anti-CD28 antibodies and treated for 72h (24h for IL-2 secretion) with 0.6 mM or 4.8 mM 2DG (denoted by shades of orange) during the activation. After treatment, the cells were harvested and spun down, and supernatant collected. The concentration of IFN-γ (**A**) and IL-2 (**B**) in supernatants were determined with ELISA. The cells were stained with antibodies and analyzed by flow cytometry. The expression levels of activation marker CD69 (**C**, **D**) and exhaustion marker/immune checkpoint PD-1 (**E**, **F**) were determined in CD4+ (**C**, **E**) and CD8+ (**D**, **F**) T cells. The individual data points represent individual donors, while the horizontal lines represent the mean ± SEM of six (**A**) or seven (**C-F**) independent experiments with different healthy donors. *p<0.05, **p<0.01 as determined by repeated measures one-way ANOVA with Dunnett's post-hoc test. (**G**) The gating strategy for the analysis of CD69 and PD-1 expression by flow cytometry. The gates for CD69+ and PD-1+ populations were set according to fluorescence minus one (FMO) controls.

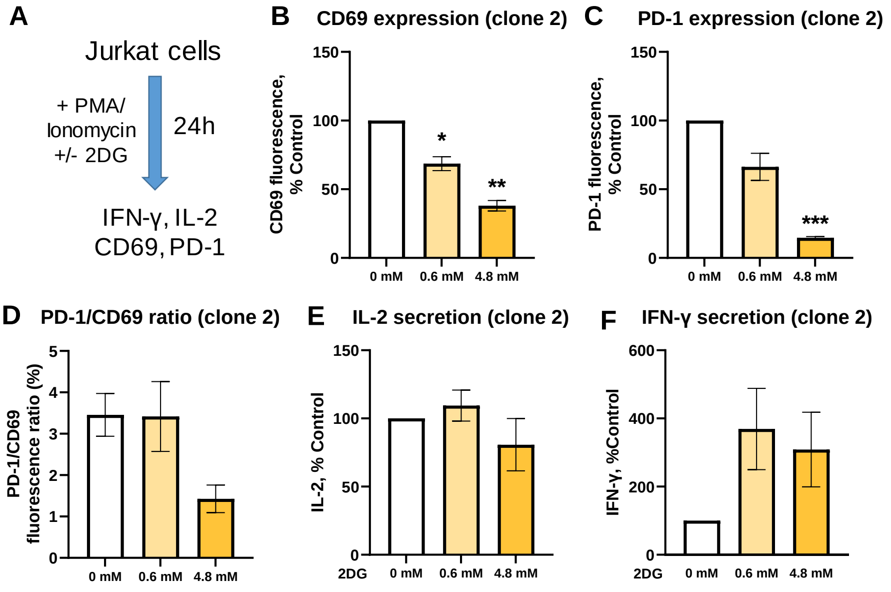

Figure S3: The effect of concurrent 2-deoxy-D-glucose treatment on IFN-γ secretion and activation markers in activated Jurkat cell clone 2. Jurkat cells not obtained directly from ATCC were activated with PMA/ionomycin and treated with 0.6 mM or 4.8 mM 2DG as indicated for 24h. (**A**) An overview of the treatment and activation protocol. (**B-D**) Relative surface expression of CD69 (**B**, **D**) and PD-1 (**C**, **D**) was determined by flow cytometry. The ratio of PD-1 and CD69 fluorescence is displayed in (**D**). (**E**, **F**) The concentration of IL-2 (**E**) and IFN-γ (**F**) in the medium was determined by ELISA. The mean ± SEM is shown for three independent experiments. *p<0.05, **p<0.01, ***p<0.001 as determined by repeated measures one-way ANOVA with Dunnett's post-hoc test.

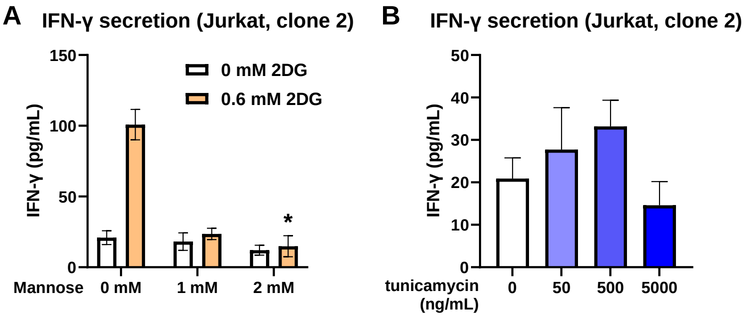

Figure S4: The role of protein N-glycosylation in the effect of concurrent 2-deoxy-D-glucose treatment on IFN-γ secretion in activated Jurkat cell clone 2. Jurkat cells not obtained directly from ATCC were activated with PMA/ionomycin and treated with 0.6 mM in the presence or absence of 1-2 mM mannose as indicated for 24h (**A**). Alternatively, Jurkat cells were activated with PMA/ionomycin and treated with the indicated concentrations of tunicamycin for 24h (**B**). The concentration of IFN-γ in the medium was determined by ELISA. The mean ± SEM is shown for two independent experiments. **p<0.01, ***p<0.001 as determined by repeated measures one-way (**B**) or two-way (**A**) ANOVA with Dunnett's or Šidak's post-hoc test.

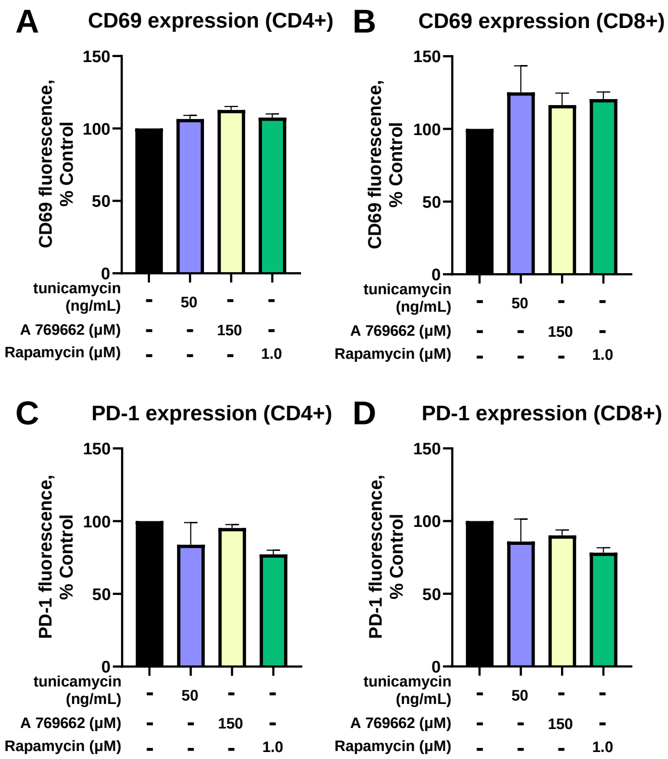

Figure S5: The effect of tunicamycin, A 769662 and rapamycin treatment on T cell activation and PD-1 expression in pre-activated T cells from PBMC. PBMC were activated with anti-CD3 and anti-CD28 antibodies for 48h, harvested and washed, then treated for 24h with tunicamycin, A 769662 and rapamycin, stained with antibodies and analyzed with flow cytometry. The expression levels of activation marker CD69 (**A**, **B**) and exhaustion marker/immune checkpoint PD-1 (**C**, **D**) were determined in CD4+ (**A**, **C**) and CD8+ (**B**, **D**) T cells. The data represent the mean ± SEM of three independent experiments with different healthy donors. The data were analyzed using repeated measures one-way ANOVA with Dunnett's post-hoc test.

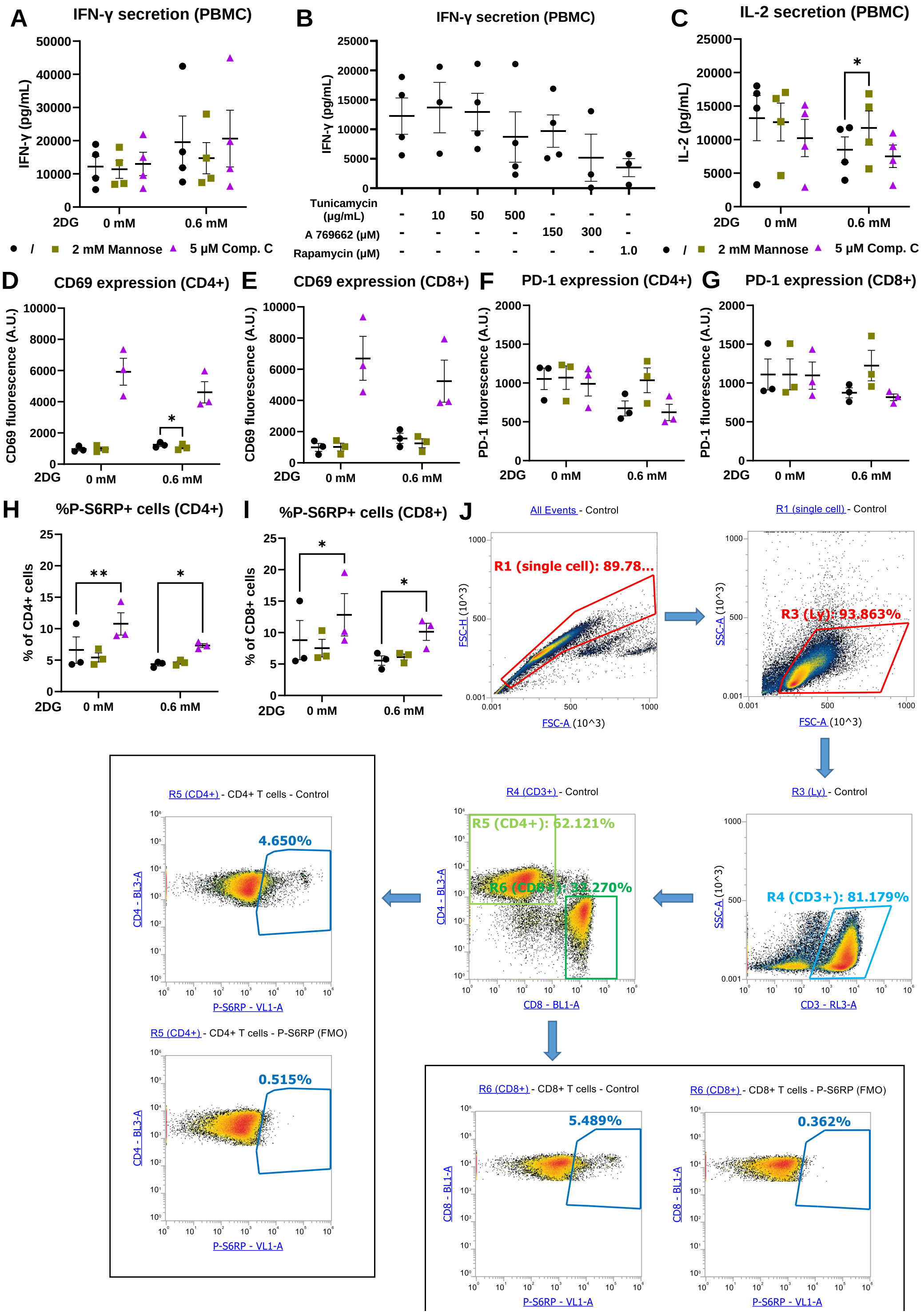

Figure S6: The role of protein N-glycosylation and AMPK activation in the effect of 2DG. PBMC were activated with anti-CD3 and anti-CD28 antibodies for 48h, harvested and washed, then treated for 24h with 0.6 mM 2DG in the presence or absence of 2 mM mannose or 5 µM compound C (following 30 min pretreatment) (**A, C-K**). Alternatively, the cells were treated after activation with tunicamycin, A 769662 or rapamycin as indicated (**B**). For cytokine secretion, the cells were restimulated with PMA and ionomycin for 4h, after which cell culture supernatants were collected and the concentration of IFN-γ (**A, B**) and IL-2 (**C**) determined with ELISA. The expression levels of activation marker CD69 (**D, E**) and exhaustion marker/immune checkpoint PD-1 (**F, G**) were determined in CD4+ (**D, F**) and CD8+ (**E, G**) T cells using flow cytometry. The phosphorylation of S6RP in CD4+ (**H**) and CD8+ (**I**) T cells was determined using intracellular staining flow cytometry. The individual data points represent individual donors, while the horizontal lines represent the mean ± SEM of three (**D-I**) to four (**A-C**) independent experiments with different healthy donors. *p<0.05, **p<0.01 as determined by repeated measures one-

way ANOVA (**B**) or repeated measures two-way ANOVA (**A, C-I**) with Dunnett's or Šidak's post-hoc test. (**J**) The gating strategy for the analysis of S6RP phosphorylation in CD4+ and CD8+ T cells from PBMC. The gates for P-S6RP+ populations populations were set according to fluorescence minus one (FMO) control.

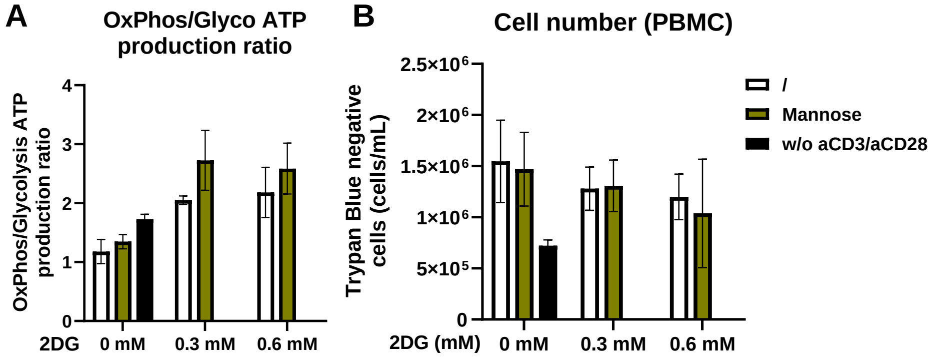

Figure S7: Energy metabolism of T cells from PBMC– the ratio between the ATP produced by oxidative phosphorylation (OxPhos) and the ATP produced by glycolysis (glyco ATP). PBMC were activated with anti-CD3 and anti-CD28 antibodies for 48h, harvested and washed, then treated for 24h with 0.3 mM or 0.6 mM 2DG. (**A**) The ATP production from oxidative phosphorylation and glycolysis were calculated according to the manufacturer's instructions for Seahorse Real Time ATP Assay. (**B**) The number of Trypan blue negative cells after 48h activation and 24h treatment is shown. Mean ± SEM is shown for three independent experiments with different donors.

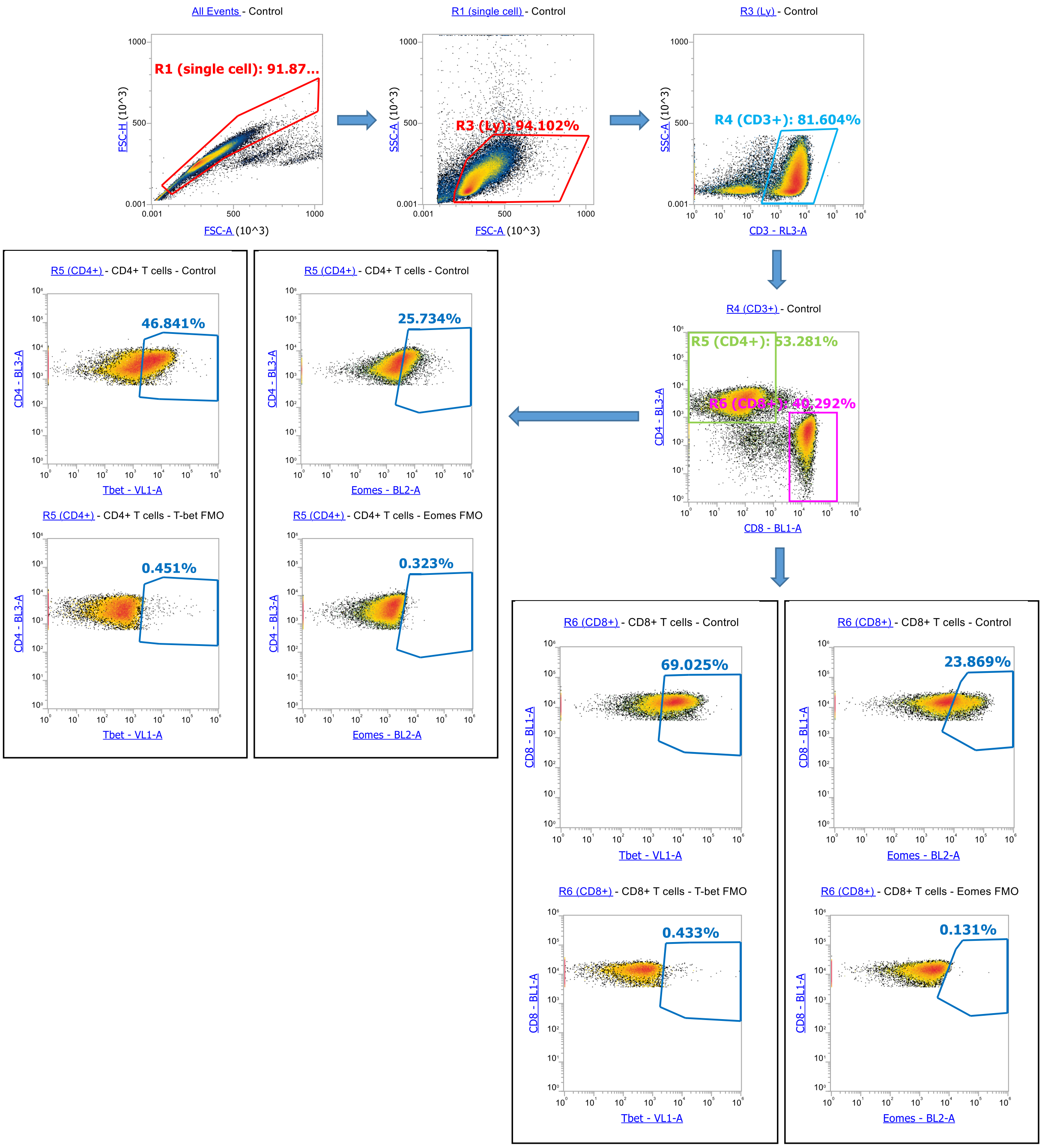

Figure S8: The gating strategy for the analysis of Tbet and Eomes expression in CD4+ and CD8+ T cells from PBMC. The gates for Tbet+ and Eomes+ populations were set according to fluorescence minus one (FMO) controls.

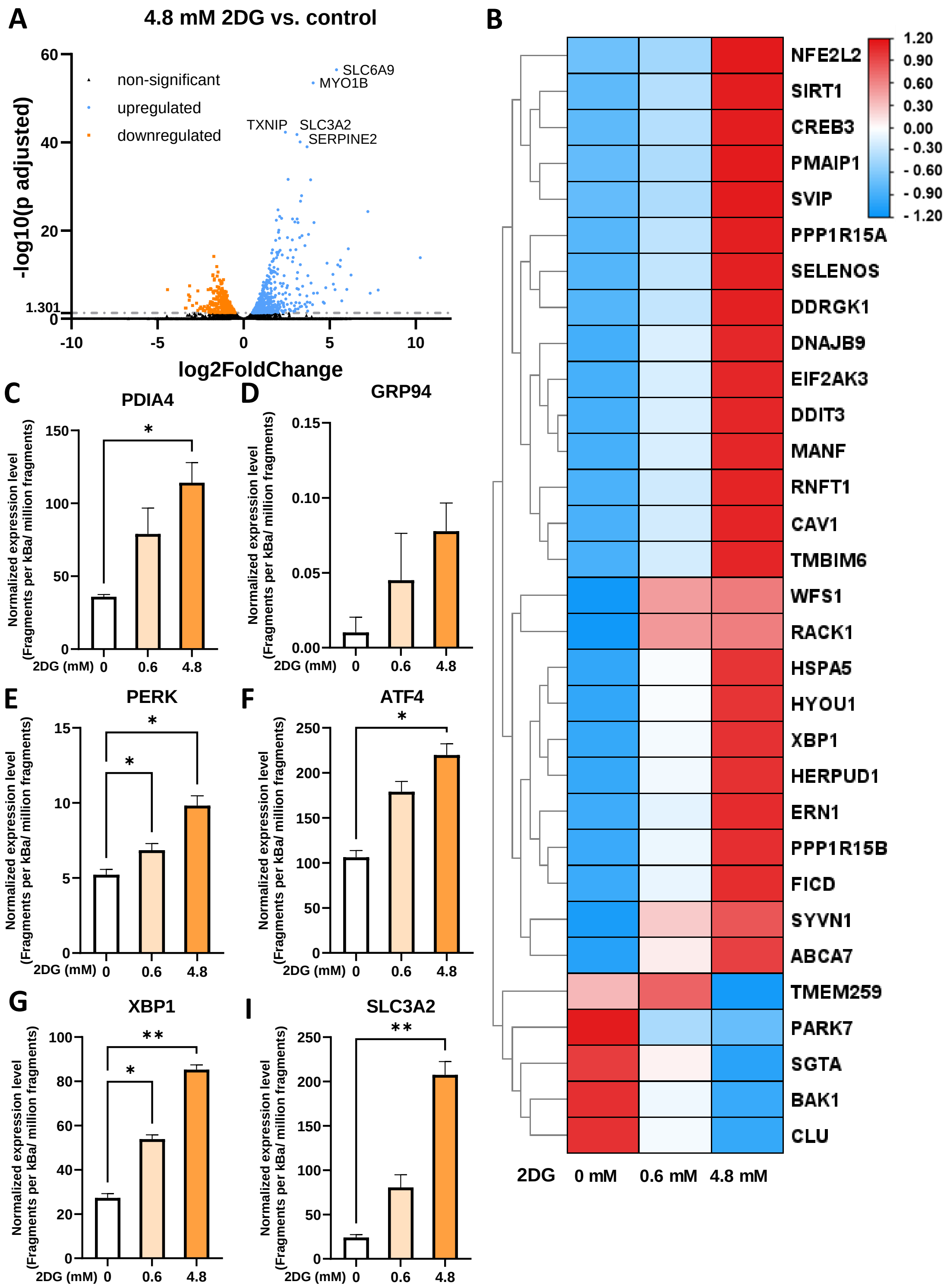

Figure S9: The transcriptomics analysis of 2DG treated T cells from PBMC. PBMC were activated with anti-CD3 and anti-CD28 antibodies for 48h, harvested and washed, then treated with 0.6 mM or 4.8 mM 2DG for 24h. After treatment, the cells were harvested, washed and snap frozen in liquid nitrogen. Total mRNA was isolated and the relative expression levels of individual mRNA determined with RNAseq. (**A**) The volcano plot of differentially expressed genes in 4.8 mM 2DG treated versus control cells with five most significantly altered genes marked. (**B**) Heat map analysis of genes involved in the ER stress. The z-score displayed on the heatmap was calculated from FPKM. (**C-F**) The relative mRNA expression levels of chaperones PDIA4 (**C**) and GRP94 (**D**), ER stress markers PERK (**E**), ATF4 (**F**) and XBP1 (**G**) as well as SLC3A2 (**H**). The data represent the mean ± SEM of three independent experiments with different healthy donors. *p<0.05, **p<0.01 as determined by repeated measures one-way ANOVA (**C-H**) with Dunnett's post-hoc test.

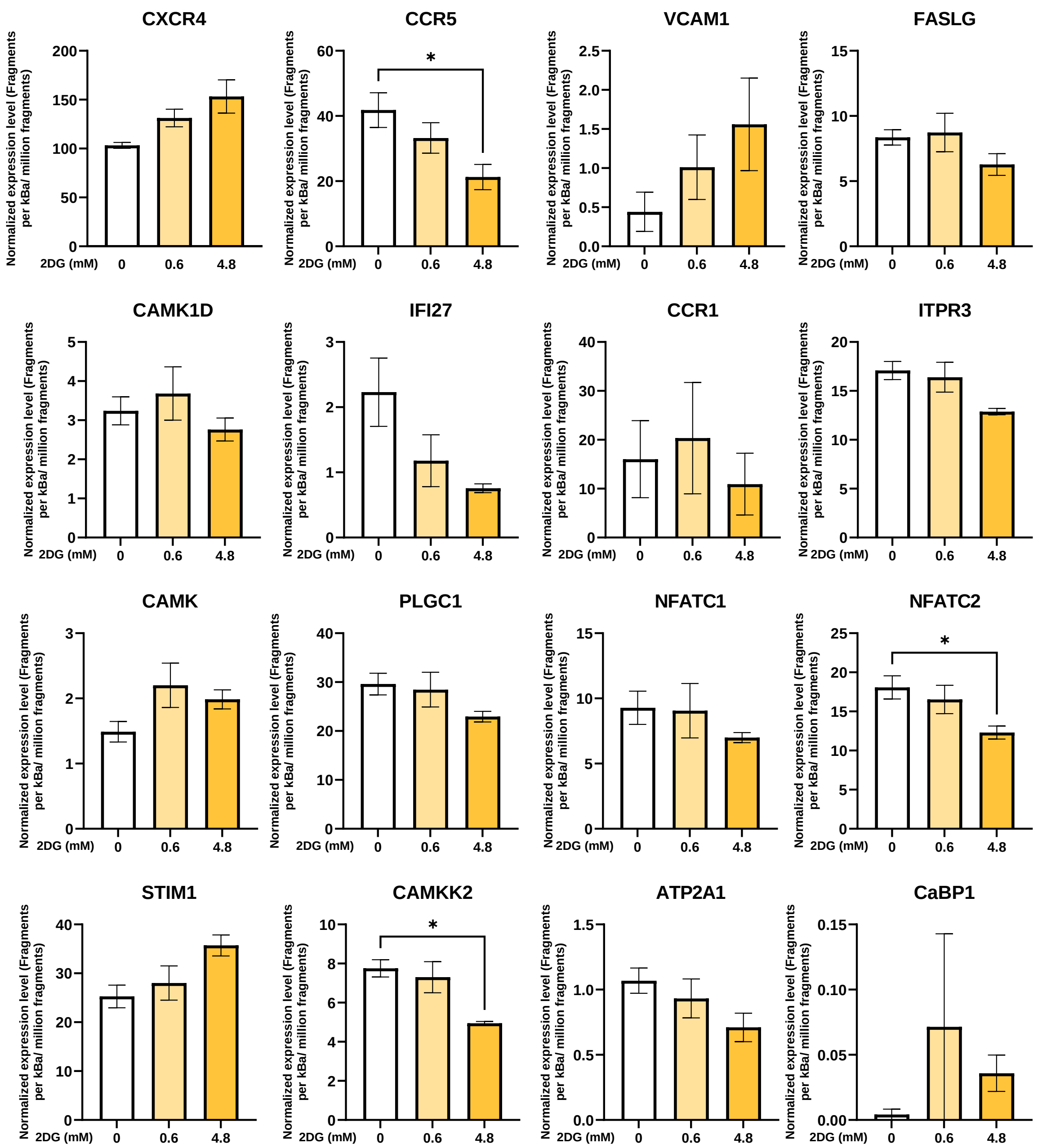

Figure S10: The transcriptomics analysis of 2DG treated T cells from PBMC for genes involved in Ca^{2+} signaling. PBMC were activated with anti-CD3 and anti-CD28 antibodies for 48h, harvested and washed, then treated with 0.6 mM or 4.8 mM 2DG for 24h. After treatment, the cells were harvested, washed and snap frozen in liquid nitrogen. Total mRNA was isolated and the relative expression levels of individual mRNA determined with RNAseq. *p<0.05 as determined by repeated measures one-way ANOVA with Dunnett's post-hoc test.

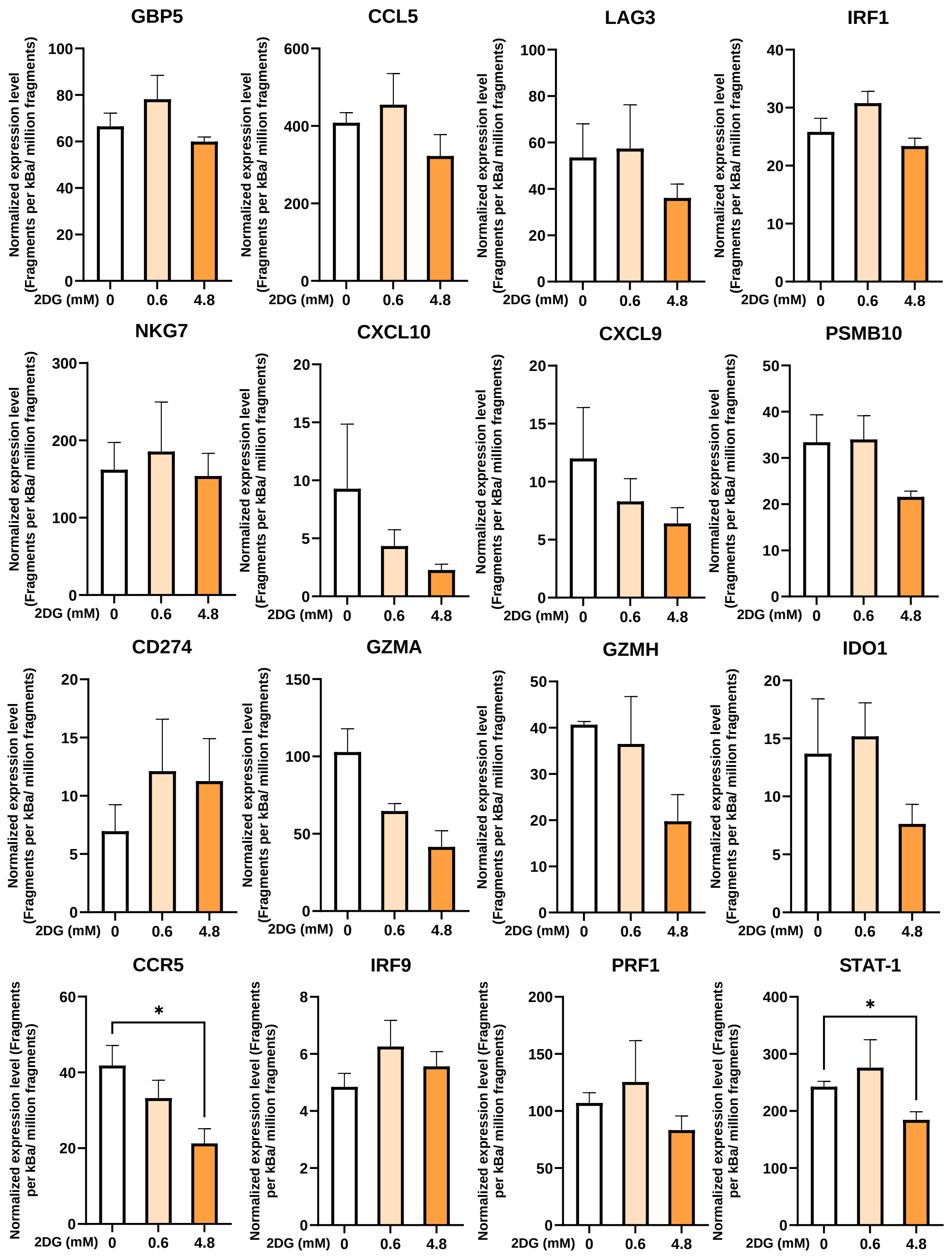

Figure S11: The transcriptomics analysis of 2DG treated T cells from PBMC for interferon-stimulated genes. PBMC were activated with anti-CD3 and anti-CD28 antibodies for 48h, harvested and washed, then treated with 0.6 mM or 4.8 mM 2DG for 24h. After treatment, the cells were harvested, washed and snap frozen in liquid nitrogen. Total mRNA was isolated and the relative expression levels of individual mRNA determined with RNAseq.

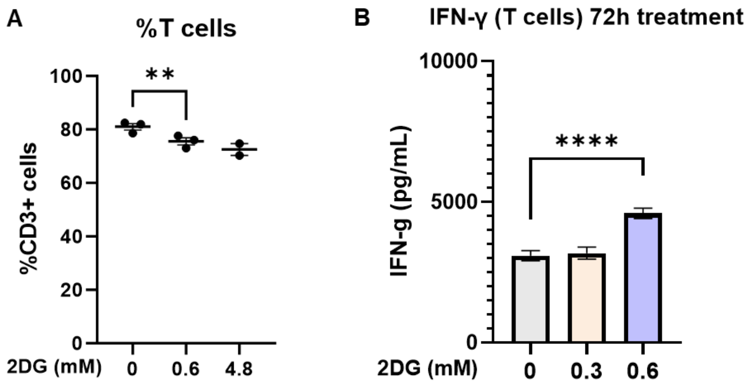

Figure S12: The effect of 2DG treatment on IFN-γ **secretion in isolated T cells from pre-activated PBMC. A)** Percentage of T lymphocytes (CD3+) in PBMC after 48h activation with anti-CD3 and anti-CD28 antibodies and 24h treatment with 2DG. The individual data points represent measurements in one donor, the mean ± SEM is presented. **B)** PBMC were washed following by isolation of T cells with the MojoSort Human CD4 T/CD8 T Cell Isolation kit (Biolegend). Isolated T cells were seeded and activated with anti-CD3 and anti-CD28 antibodies for 72h. The T cells were treated for 72h with 0.3 mM or 0.6 mM 2DG. After 72h T cells were restimulated with PMA and ionomycin for 4h, after which media supernatants were collected. The concentration of IFN-γ in supernatants were determined with ELISA. Presented are measurements in one donor, mean and ± SEM of the technical repeats are shown, ****$p<0.0001$ as determined by paired t-test (two-tailed).

Supplementary methods

Flow cytometry gating strategy

For experiments with PBMC, doublets were first excluded from further analysis by gating on the forward scatter area vs. height plot. Next, dead cells were excluded from analysis by excluding Zombie Yellow high cells. The lymphocyte population was gated on the FSC/SSC plot, with the lymphocyte gate confirmed by a separate sample stained for CD14 to ensure minimal inclusion of monocytes in the analysis. The lymphocyte population was then gated on CD3+ cells, followed by separate CD4+ and CD8+ gates, on which the final analysis was conducted. The gates for CD69+, PD-1+, P-S6RP+, Eomes+ and Tbet+ populations was determined using appropriate fluorescence minus one (FMO) controls.
